# Passive Sampling Helps the Appraisal of Contaminant Bioaccumulation in Norwegian Fish Used for Regulatory Chemical Monitoring

**DOI:** 10.1021/acs.est.2c00714

**Published:** 2022-06-07

**Authors:** Ian John Allan, Branislav Vrana, Anders Ruus

**Affiliations:** † Norwegian Institute for Water Research, Økernveien 94, Oslo NO-0579, Norway; ‡ RECETOX, Faculty of Science, Masaryk University, Kotlarska 2, Brno 61137, Czech Republic

**Keywords:** passive sampling, hexachlorobenzene, biota, fish, water framework directive, polychlorinated biphenyls

## Abstract

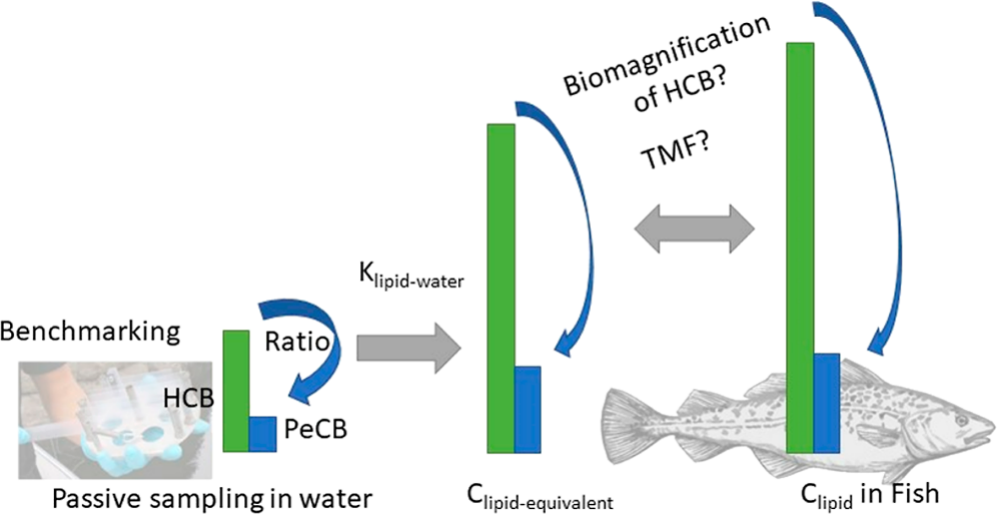

Hexachlorobenzene (HCB), listed on the Stockholm Convention on persistent organic pollutants and regulated as a hazardous priority pollutant by the Water Framework Directive (WFD), is ubiquitously distributed in the environment and assumed to mildly biomagnify in aquatic foodwebs. The proposal to include trophic magnification factors (TMFs) in the procedure for comparing contaminant levels in biota at different trophic levels (TLs) with WFD environmental quality standards requires adequate selection of TMFs. In the first step of our study, we compared two independently obtained datasets of pentachlorobenzene (PeCB) and HCB concentration ratios from passive sampling (PS) in water and in fish through routine monitoring programs in Norway to evaluate possible biomagnification. In this procedure, PeCB is used for benchmarking the bioconcentration in fish, and the observed HCB/PeCB ratios in fish are compared with ratios expected in the case of (i) HCB bioconcentration or (ii) biomagnification using published TMF values. Results demonstrate that it is not possible to confirm that HCB biomagnifies in fish species that would be used for WFD monitoring in Norway and challenges the proposed monitoring procedures for such compounds in Norwegian or European waters. In the second step, fish-water chemical activity ratios for HCB and PeCB as well as for polychlorinated biphenyls where biota and PS were conducted alongside were calculated and found to rarely exceed unity for cod (*Gadus morhua*), a fish species with a TL of approximately 4.

## Abstract 2

Compliance monitoring for contaminant concentrations in fish requires robust trophic magnification factors. Passive sampling and benchmarking provide evidence or lack of evidence of biomagnification in Norwegian fish used for monitoring.

## Introduction

Chemical monitoring in fish is proposed to evaluate the level of selected persistent hydrophobic and nonionized contaminants in water bodies across Europe in response to Water Framework Directive (WFD) legislation.^1−4^ Environmental quality standards (EQSs) have been derived to protect from adverse effects of these chemicals to aquatic organisms and potential for secondary poisoning. This proposal for use of biota for monitoring causes challenges related to the selection of the most appropriate species, trophic level (TL), size, age, sex, and matrix for analysis, some of which are yet to be addressed. In Norway, coastal monitoring uses cod (*Gadus morhua*) and analysis of priority pollutants in fish liver. Freshwater biomonitoring relies mostly on analysis in salmonids (brown trout and salmon). Because not a single species at one specific TL can be found in all water bodies, the proposed EQS values correspond to a hypothetical fish at a TL of 5 for the marine environment and 4 for freshwaters.^5^ Despite the relative scarcity of published trophic magnification factors (TMFs) for WFD priority substances, they are proposed as a means to adjust the observed levels in fish to a TL adequate for comparison with EQS.^1,3,5,6^ This means a clear understanding of bioaccumulation and associated uncertainties is compulsory for the application of such procedures in regulatory settings. Passive sampling (PS) for nonionized hydrophobic chemicals with absorption-based passive samplers is increasingly being used to help understand bioaccumulation.^7−11^ PS can be used for example to estimate freely dissolved contaminant concentrations (*C*_free_) in waters biota were exposed to, and to calculate in-situ bioconcentration or bioaccumulation factors (BCFs or BAFs).^7,12^ Recently, improvements in the comparison of chemical activities of contaminants in aquatic organisms with those in the surrounding abiotic environment have been made using partitioning PS techniques.^13−15^ With the help of contaminant PS to silicone rubber (SR) and available lipid-SR partition coefficients (*K*_lip-sr_), it is possible to calculate contaminant concentrations in lipid that would be at equilibrium with the water (*C*_lip,equiv_) phase in the investigated water body.^9^ This in turn can be used for comparing the contaminant levels in water and fish with the same units, that is, ng g^–1^ lipid. While this has been undertaken with fish and passive samplers equilibrated with the bottom sediment from where the fish were sampled,^16−18^ it has seldom been done with passive samplers exposed to water.^9^

The aim of this study was to re-enforce the interpretation of contaminant bioaccumulation in fish from the Norwegian environment with PS through activity ratios and benchmarking against data for a chemical not expected to biomagnify in fish [pentachlorobenzene (PeCB)]. We have combined biomonitoring with cod (*G. morhua*) for coastal and marine sampling locations and a range of freshwater fish including brown trout (*Salmo trutta*), Atlantic salmon (*Salmo salar*), European perch (*Perca fluviatilis*), and Arctic char (*Salvelinus alpinus*) for river and lake biomonitoring with a decade of PS in Norway.^19^ Second, we investigated activity ratios for polychlorinated biphenyls (PCBs) in cod from sites at which biomonitoring and PS were conducted alongside.

## Methods

### Freshwater Biomonitoring Data

We re-investigated the data we obtained from river Alna where contaminant accumulation in 28d-caged brown trout was undertaken alongside PS in the river and in vivo in fish.^11^ We then searched for data for freshwater fish and collated these data through the Norwegian environmental contaminants database (https://vannmiljo.miljodirektoratet.no/, accessed from 06-2019/06-2020). While much data for hexachlorobenzene (HCB) exist, PeCB data remain scarce either because the chemical was not monitored for or because of poor limits of quantification (LOQs). Brown trout (*S. trutta*) and salmon (*S. salar*) data were obtained for five rivers sampled in 2018 from the “Elveovervåkingsprogrammet” (Table SI-1).^20^ Data were available for brown trout, European perch, and Arctic char for 2015, 2017, and 2018 through the “Milfersk” sampling program for approximately 30 lakes across Norway (Table SI-2).^21^ Data for an additional 14 lakes from Lyche et al. (2019) where brown trout, arctic char, perch, and whitefish were sampled in 2018 were included (Table SI-3).^22^

Analysis for the different studies was undertaken either from individual or composite whole fish samples or individual or composite of fillets or livers. The extractable organic matter (EOM) content was also measured in most cases and reported as lipid content of the fish samples analyzed. In a minor number of cases, PeCB concentrations for individual fish or composite fish samples were below the LOQ, often for samples with low lipid content and where HCB concentrations were low. Because of the limited occurrence in the overall dataset, we decided not to use these further.

### Marine Biomonitoring Data

Biomonitoring data were obtained from the Norwegian environmental contaminants database and/or monitoring reports by NIVA. Cod data for 2009–2011 from Andøya, Jan Mayen, Svalbard, and Bear Island were obtained from the “Tilførelsprogrammmet” reports (Table SI-4).^23−25^ Data from the “Milkys” program of monitoring (2013–2016) were for the following sampling sites: Oslofjord, Kvænangen, Kristiansand, and Egersundbanken.^26−28^ Additional Oslofjord data (2015–2016) were from the “Forsuringsovervåking” and “Miljøgifter i en urbanfjord” monitoring programs.^29^

In most cases, 15–25 organisms were fished per sampling location. Fish livers or fillet were dissected and homogenized prior to extraction and analysis. EOM was also measured. A number of laboratories were involved in the analyses for the whole dataset used here. We assume laboratories use similar/consensus methods for lipid content estimation, and the relative variability resulting from EOM measurement is low. Incidentally, the lipid content does not intervene in the benchmarking when using lipid-based fish concentrations since it appears both in the numerator and denominator of eq 4.

### PS Data

The SR PS data reported previously^19,23,30,31^ included freshwater and marine sites in Norway sampled in the period 2009–2019. Marine monitoring sites where PS and chemical monitoring in fish were conducted alongside for HCB and PeCB included Jan Mayen, Bear Island, Andøya, Kristiansand, and Oslofjord. Fish and PS were undertaken in close proximity and were overlapping in time. For HCB only, corresponding PS and biomonitoring data also exist for Hvaler and Ålesund monitoring sites. Sampler deployment times varied from weeks/months to an entire year for selected sites. For PCBs, datasets of combined cod and PS included all sites above, but the highest amounts of data were for Hvaler, Oslofjord, and Ålesund with samplers deployed on a yearly basis over a period of 4 years.^31^ Here, the average of *C*_free_ for HCB, PeCB, and PCBs is for duplicate samplers deployed each year at each site (Table SI-5). Tables SI-6 and SI-7 report mean values of *C*_sr equil_ and *C*_lip,equiv_.

The NIVA laboratory performing the SR preparation, extraction, and analyses participated in all rounds of Quasimeme proficiency testing schemes with suitable performance for these chemicals.^32^

### Activity Ratios and Benchmarking

The ratio of activity in fish (*A*_Fish_) over that in water (*A*_water_) can be calculated with the following equation
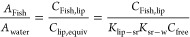
1where the activity in fish is represented by the contaminant concentration in the fish or fish tissue on a lipid basis (*C*_Fish,lip_) and the activity in water by *C*_lip,equiv_ calculated from *C*_free,_ the SR-water partition coefficient (*K*_sr-w_), and *K*_lip-sr_. The product of *K*_lip-sr_ and *K*_sr-w_ is a lipid-water partition coefficient (*K*_lip-w_) that can be interpreted as a hypothetical lipid-based BCF, equivalent to a BCF for small fish or primary consumer in the absence of metabolism of the chemical. A ratio of 1 can be expected for a contaminant for which the concentration in the fish is close to equilibrium with that in water and for which partitioning to lipids is the main mechanism of bioaccumulation. A ratio well above 1 can be expected for a chemical that undergoes biomagnification. The *C*_lip_ in organisms at different TLs is connected through the TMF and the following equation.
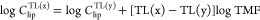
2

For a chemical such as HCB expected to biomagnify to a mild extent, a TMF of 3.4^3,33^ corresponds to a ratio of activities ∼50 (eq 1) for a fish with a TL of 4.

PeCB on the other hand with TMF <1 is not expected to biomagnify in aquatic foodchains.^33^ However, with a log *K*_ow_ of about 5, a high persistence, and its ubiquitous distribution across the European environment, it is a prime candidate for benchmarking. Benchmarking to evaluate bioaccumulation has been done before.^34^ If *K*_lip-sr_ and *K*_sr-w_ are known for PeCB and for substance x, it is possible to calculate a hypothetical ratio of *K*_lip-w_, or in other words, the ratio of concentrations that can be expected if the bioconcentration is solely responsible for bioaccumulation of both substances and they have the same *C*_free_
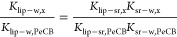
3

When *C*_free_ of both substances in water are measured with SR PS, the ratio of *C*_lip,equiv_ can be estimated (eq 1) and compared with the actual ratios in fish
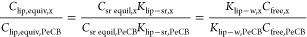
4where *C*_sr equil_ is the concentration in SR at equilibrium with that in water. In eq 4, a lower ratio may indicate that active selective elimination processes such as metabolism or excretion reduce the concentration of substance x in fish, while a higher ratio could indicate a tendency to biomagnify.

### Calculation of Expected *K*_lip-w_ and BAFs

Over the last decade, an increasing number of measurements of lipid-polymer partition coefficients has been undertaken for different polymers and lipid types. The variability of *K*_lip-sr_ for different lipid types is very low.^13−15^ For HCB and PeCB, we have used log *K*_sr-w_ of 4.6 and 5.1 from Smedes et al. (2017)^15^ and *K*_lip-sr_ of 7.38 and 9.35 g g^–1^ for AlteSil SR (Tables 1 and SI-8). Confidence intervals (95%) of the measurements of log *K*_sr-w_ for these two compounds were 0.05 and 0.06 of log unit,^35^ indicating that the error on these *K*_sr-w_ will have a minor impact on the ratio of *K*_lip-w_ for HCB/PeCB. Partitioning to SR is expected to increase with decreasing temperature leading to the possible need to use temperature-corrected *K*_sr-w_ in the estimation of *C*_free_. However, both BAFs and *K*_lip-w_ (e.g. in Eq 4) would be expected to increase in this situation too since most of the increase is related to the decrease of solubility in water. We therefore decided not to apply data corrections for temperature or salinity effects. According to eq 3, we calculated a *K*_lip-w_ of 307 650 and 1049087 L kg^–1^ for PeCB and HCB with a factor of 3.4 between the two. This value is in agreement with the values reported by Adolfsson-Erici et al. (2012)^34^ and Inoue et al. (2012)^36^ for rainbow trout and common carp. When comparing with BCFs, our calculated log *K*_lip-w,HCB_ is at the top of the range of observed log BCF of 3.57–4.70 compiled by Arnot and Gobas (2006).^37^ While PeCB is not expected to biomagnify in an aquatic foodweb,^33^ TMF values in the range of 1.7 to 4.75 with an average of 3.4 for HCB have been used or reported.^3,33,38−40^ We then calculated the relative difference in hypothetical BAFs for these two compounds for fish with a TL of 3–4 and TMFs above.
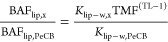
5

**Table 1:** Expected BAF_lip,HCB_/BAF_lip,PeCB_ and *C*_lip,HCB_/*C*_lip,PeCB_ Ratios Calculated for Freshwater and Marine Fish at a TL of 3–4 and for an Average TMF of 3.4 (Range of 2–4) for HCB

			BAF_lip,HCB_/BAF_lip,PeCB_^c^		*C*_lip,HCB_/*C*_lip,PeCB_^d^	
	*C*_free,HCB_/*C*_free,PeCB_^a^	*K*_lip-w,HCB_/*K*_lip-w,PeCB_^b^	TL = 3	TL = 4	TL = 3	TL = 4
freshwater	3.93	3.4	39 (14–55)	134 (27–218)	155 (53–214)	525 (107–855)
marine	2.76	3.4	39 (14–55)	134 (27–218)	108 (37–150)	369 (75–600)

a Ratio of freely dissolved concentrations as observed in water bodies without specific contaminant with either of the chemicals.

b Ratio of *K*_lip-w_ calculated independently from *K*_sr-w_ and *K*_lip-sr_.

c Ratio of hypothetical BAFs calculated for fish with a TL of 3–4 and for the average of reported TMF values for HCB (see text) assuming no biomagnification of PeCB (eq 5).

d Ratio of hypothetical lipid-based fish concentration calculated for fish with TL of 3–4 and for the average of reported TMF values for HCB (see text) assuming no biomagnification of PeCB.

The ratio of BAF_lip,HCB_/BAF_lip,PeCB_ increases from the ratio of *K*_lip-w_ of 3.4 to values on average of 39 and 134 for TLs of 3 and 4, respectively (Table 1). For this calculation, we have assumed that bioconcentration is generally representative of species at a TL of 1. We also estimated the ratio of hypothetical lipid-based concentrations for compound x and PeCB for fish at a specific TL
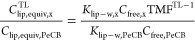
6

Considering a background *C*_free,HCB_/*C*_free,PeCB_ ratio of 3.93 and 2.76 for freshwater and marine waters, the overall ratios of HCB/PeCB concentrations in fish would be on average 155 and 525 for freshwater fish with a TL of 3 and 4, respectively (Table 1). Lower ratios of 108 and 369 can be expected in the marine environment.

## Results and Discussion

### Caged brown Trout in River Alna

**Figure 1:**
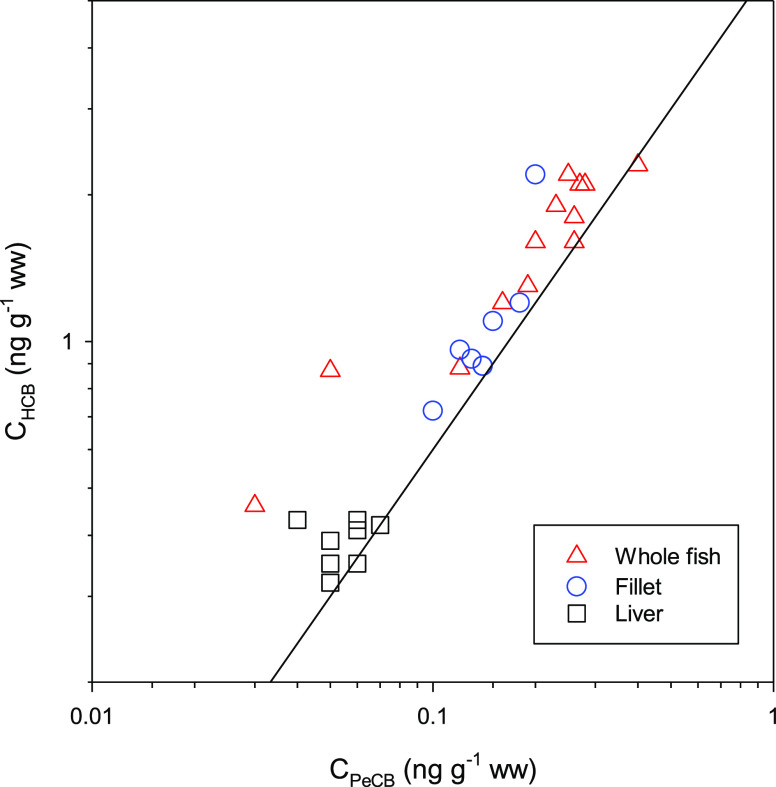
Concentrations of PeCB and HCB in whole organisms, fillet, and liver of caged brown trout (*S. trutta*) in the Alna river in Oslo.^11^ The slope of the solid line represents the ratio of abiotic *C*_free_ × *K*_lip-w_ for HCB over PeCB.

The fish were not fed during the caging experiment in river Alna,^11^ and therefore bioconcentration was expected to be the main mechanism of uptake for both compounds with a theoretical ratio of *K*_lip-w_ of 3.4 (Table 1). Taking into account *C*_free_ for HCB and PeCB in the river, relative concentrations that could be expected in fish tissues at equilibrium with the water phase were calculated and are represented by the linear relationship given in Figure 1. The slope is the nominal ratio of HCB and PeCB expected in fish tissues at equilibrium with the water. HCB and PeCB data for individual fish, either whole fish, fish fillet, or liver, are compared with this relationship. Except for a few whole fish and liver samples, most datapoints fall very close to the line. Since the fillet represents a significant proportion of the fish in mass, it is not surprising to observe wet weight concentrations of HCB and PeCB for whole fish and fillet muscle in the same range on Figure 1. Concentrations in the liver also fall onto the reference line. In this example, if processes other than bioconcentration were involved in the bioaccumulation of these two compounds, we could expect concentrations to deviate strongly from the reference line. The scatter of the data in the lower left-hand corner may be due to increased uncertainty of the analysis at concentrations closer to LOQ. Biomagnification of HCB only would shift all datapoints toward the top right corner of the graph. Overall, these data indicate a similar chemical activity of these contaminants in fish and in the water they have been exposed to and that processes such as metabolism, if occurring, have only a minor influence on levels in brown trout.

### HCB and PeCB in Freshwater Fish in Norway

**Figure 2:**
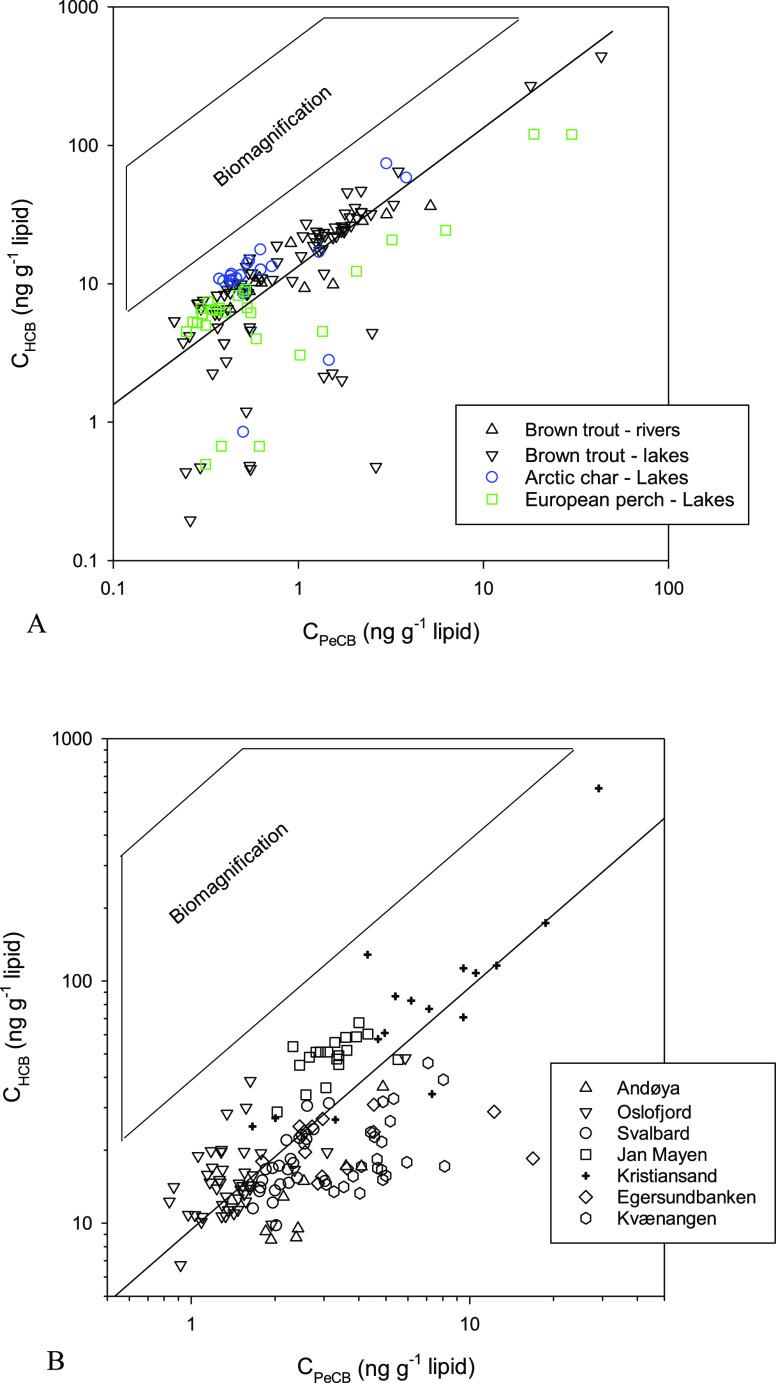
Lipid-based concentrations of PeCB and HCB in freshwater fish (A) from Norwegian lakes and rivers (*n* = 167) in cod liver (B) from different sites in Norwegian marine waters. The slope of the solid line represents the ratio of abiotic *C*_free_ × *K*_lip-w_ for HCB over PeCB. The slopes of the lines forming the “biomagnification” box represent the expected HCB/PeCB concentration ratio in the hypothetical case of TMF for HCB of 3.4 for organisms with a TL of 3 or 4 for freshwaters and a TL of 4 for marine waters, assuming PeCB only bioconcentrates.

Since fish biomonitoring is principally used for monitoring, freshwater fish species used include arctic char, brown trout, European perch, and in some cases salmon. Fish concentrations for HCB and PeCB compiled from the Norwegian environmental contaminant database, plotted against each other, are compared with a reference line representing the bioconcentration of the two chemicals in Figure 2A. Considering the theoretical *K*_lip-w,HCB_/*K*_lip-w,PeCB_ ratio of 3.4 and the empirical ratio of *C*_free_ of the two chemicals of 3.93 in unimpacted freshwaters, the slope of this reference line is 13.4. As can be seen in Figure 2A, most data generally follow the reference line, on or slightly above it. This is generally irrespective of the concentration level of these compounds that can span over 3 orders of magnitude. However, the difference in HCB and PeCB concentrations in fish never appear to exceed the reference line sufficiently to be interpreted as biomagnification of HCB. Considering a likely TL of 3 to >4 for Arctic char, brown trout, or perch and a TMF of 2–4 for HCB, a *C*_lip,HCB_/*C*_lip,PeCB_ in the range of 53 to 855 could be expected (Table 1).^21,41^ As shown in Figure 2A, none of the data reach this level. The median *C*_lip,HCB_/*C*_lip,PeCB_ ratio for the entire dataset (*n* = 167) is 17.4. This corresponds to a *C*_free,HCB_/*C*_free,PeCB_ ratio of 5 rather than 3.93 which is not unrealistic. It also corresponds to a TMF of 1.1 for a fish at TL = 4 and 1.15 for a fish at TL = 3. This generally confirms the low potential for biomagnification of HCB in freshwater fish. Literature values of TMF in the Han river in Korea showed some spatial and seasonal variability.^42^ TMFs for HCB and PeCB were in the range of 1.26–2.37 and 0.66–1.54, respectively, and only one TMF for HCB of 1.26 for one location in April appeared significant. Our data are generally in line with these data. Smedes et al. (2020) also found low or negligible biomagnification of HCB and PeCB in freshwater fish at three sampling locations in central Europe.^9^ Lipid-based concentrations in fish at a different TL were very similar for PeCB and in close agreement with concentrations that would be found in lipids at partitioning equilibrium with water. This also confirms the bioconcentration for PeCB in fish with TLs between 2 and 4. For some datapoints, PeCB was below LOQ. Actual HCB/PeCB ratios in these conditions were higher than the ratios based on LOQ (average of 9.0 and spanning 1.9–19.4).

For this comparison, we have not used river or lake-specific *C*_free_ ratios, and a slight underestimation of the *C*_free_ ratio could lead to a *C*_lip,HCB_/*C*_lip,PeCB_ representative of TMF slightly above 1. It is also relatively surprising to observe for a major proportion of the data on Figure 2; these follow the reference line independently of the concentration level. In the case of a contaminated site with one of the two compounds, the altered *C*_free_ ratio would impact the ratio of fish concentrations accordingly (see eq 3). This may be the case for some of the perch data on Figure 2A. For perch data with *C*_lip,PeCB_ > 1 ng g^–1^, HCB concentrations appear correspondingly lower and may be indicative of PeCB contamination. No sites appear to show specific HCB contamination. On the contrary, some datapoints show clearly low levels of HCB, while the PeCB data remain in the range of most other sites. One possibility to explain that, other than analysis, is a lack of equilibrium or steady-state conditions in the accumulation of HCB in fish.

### HCB and PeCB in Atlantic Cod in Norway

A number of studies and monitoring programs have been conducted on the coast of Norway and have resulted in the simultaneous measurements of HCB and PeCB in Atlantic cod. Considering, as we did for freshwaters, that the HCB/PeCB ratio of *C*_free_ does not vary much over the timescale of a decade,^19^ we were able to compile cod data from 2009 for a certain number of locations along the coast and North Atlantic (Jan Mayen). The overall number of datasets is limited, mostly because of a lack of measurements of PeCB. The empirical mean ratio of *C*_free,HCB_/*C*_free,PeCB_ of 2.76 measured for marine water is lower than that for freshwaters (Table 1) and results in a reference line with a lower slope (9.4 instead of 13.4). As for freshwater fish, we compared cod concentrations with the reference line indicative of bioconcentration for the two chemicals (slope of 9.4) on Figure 2B. We can observe a larger spread of the data around the reference line. However, when considering the independent nature of the comparison, the agreement is excellent. Considering that a TL ∼ 4 is generally attributed to cod and a TMF in the range 2–4, we estimate BAF_lip,HCB_/BAF_lip,PeCB_ in the range of 27–218 and a corresponding *C*_lip,HCB_/*C*_lip,PeCB_ ratio of 75–600 for HCB biomagnification. While some data are above the reference line (Figure 2B), levels remain well below those expected for biomagnification of HCB with a TMF of 2. For datapoints with PeCB below LOQ, actual HCB/PeCB ratios in these conditions were higher than the ratios based on LOQ (average of 8.3 and spanning 1.9–21).

Some data tend to stand out in Figure 2B. The data labelled “Kristiansand” include cod from locations in the vicinity of the town of the same name and known sites of industrial contamination with chlorinated compounds. It is therefore not surprising to observe the highest lipid-based cod concentrations of HCB and PeCB for this site. While two datapoints for this location are closest to exhibit an HCB/PeCB ratio indicative of biomagnification, it could also be the result of higher HCB concentrations. Remaining datapoints are close to the reference line. A second site that stands out is Jan Mayen with much of the data grouped and consistently above the reference line. This may indicate mild biomagnification of HCB, particularly since these fish may have a diet that differ much from coastal cod, perhaps including capelin. Last, a few datapoints for Egersundbanken and Kværnangen deviate significantly from the reference line with relatively high PeCB levels, which may indicate fish living in an area with elevated concentrations of PeCB. In general, it remains impossible through this comparison to confirm that HCB biomagnifies in cod on the Norwegian coast.

**Figure 3:**
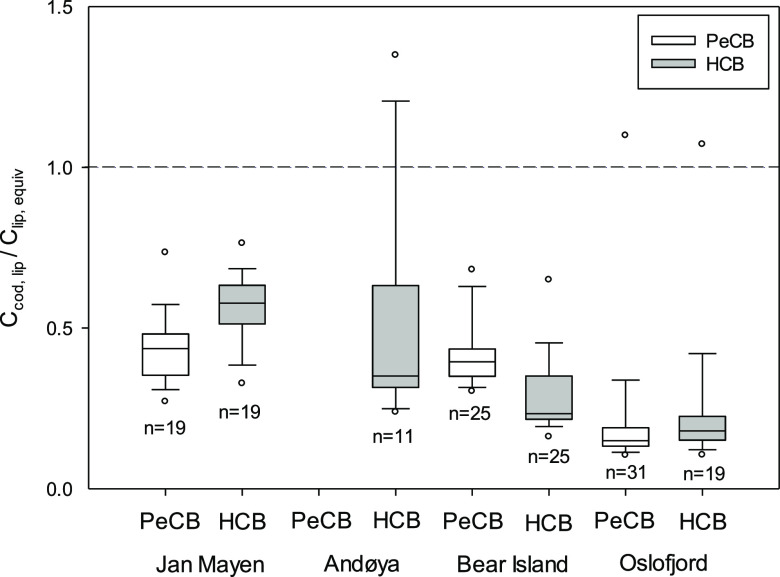
Comparison of the average chemical activity of HCB and PeCB in cod, *G. morhua* (expressed as ng g^–1^ lipid), and water (ng g^–1^ lipid at equilibrium with the water) for fish/PS sites at Andøya, Bear Island, Jan Mayen, and Oslofjord. The activity in water also corresponds to the product of *K*_lip-w_ and freely dissolved concentration in water.

For a limited number of sampling locations, namely, Andøya, Bear Island, Jan Mayen, and Oslofjord, it was possible to compare HCB and PeCB levels in cod with those in the water obtained from PS in waters in the vicinity of the cod sampling sites. The reference line in Figure 3 represents equal chemical activity in water and in fish. Lipid-based concentrations in fish liver were divided by the mean of the PS data for each site (*n* on the figure is shows the spread of the fish data). Most of the data below the reference line indicate that the chemical activity in fish never exceeds the chemical activity in water. The biomagnification of HCB in cod would be expected to result in a chemical activity significantly higher than that in water, which is not shown here. The chemical activity in fish rarely exceeds half of the activity in water. Activity ratios for PeCB at the Bear Island site are based on LOQ in fish since all PeCB concentrations in fish were below these. Real activity ratios for this site are therefore even lower. For Andøya, ratios could not be calculated for PeCB since it was not detected in passive samplers. Concentrations in fish for this site were very close to LOQ.

This divergence in activity ratios has been shown for PCBs in freshwater fish but not for marine fish and has been attributed to the slow kinetics of transfer of these chemicals from water to organisms at the base of trophic food chains.^9^ Past experiments to assess the bioaccumulation of PCBs in cod exposed to sediment/organisms from Oslofjord demonstrated generally low accumulation rates.^43^ It may be that temperature and low feeding rates may play a role here. The main lipid class in cod liver being triacyl glycerols, the use of triacyl glycerol-SR partition coefficients measured for HCB^14^ instead of generic *K*_lip-sr_ has only a minor effect on the estimated *C*_lip,equiv_.

### PCBs in Cod in Norway

**Figure 4:**
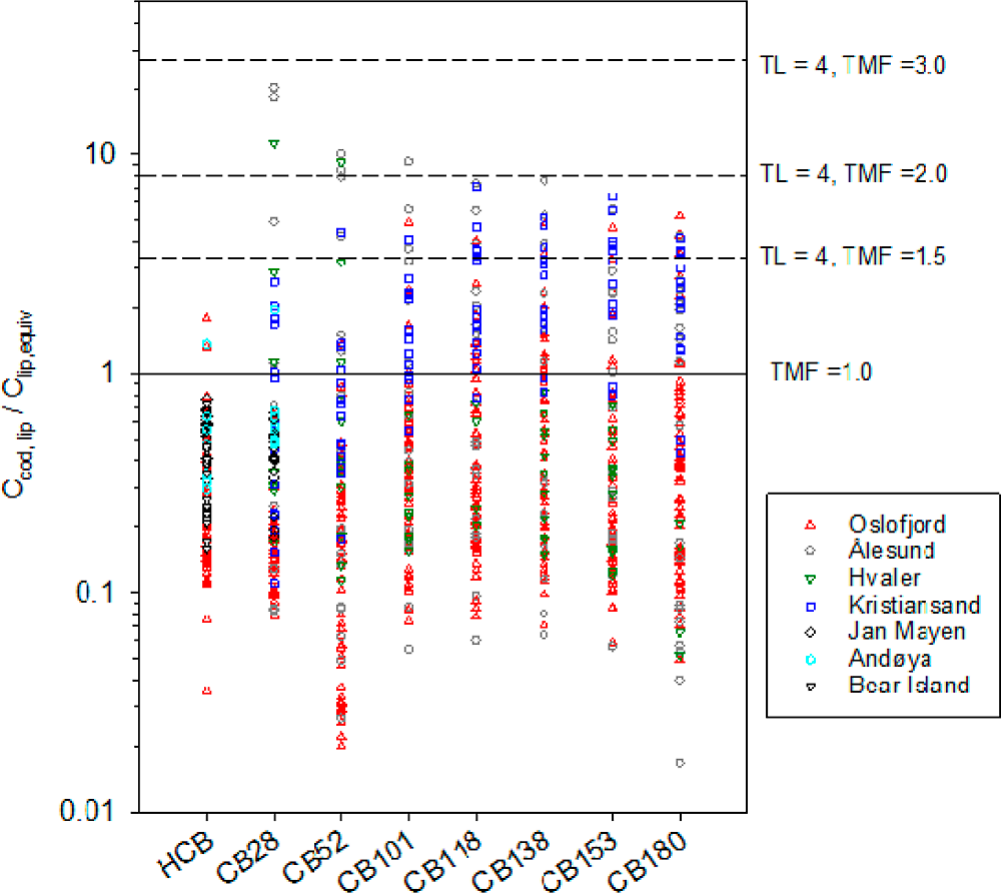
Comparison of the chemical activity of HCB and PCB congeners in cod, *G. morhua* (expressed as ng g^–1^ lipid) and in water (ng g^–1^ lipid at equilibrium with the water) for fish/PS sites at Andøya, Bear Island, Jan Mayen, Hvaler, Kristiansand, Oslofjord, and Ålesund. The activity in water also corresponds to the product of an abiotic BCF and freely dissolved concentration in water.

Cod liver has been the matrix of choice for chemical monitoring of PCB levels in Norwegian coastal waters for many years principally as a result of the widespread distribution of this species along the entire coast and the size and high lipid content of the liver rendering extraction and analyses possible. Considering the cod TL and the lipophilic properties of PCBs, biomagnification of certain congeners may be expected. The ability of PCBs and particularly of congeners with a high degree of chlorination to biomagnify in aquatic foodwebs is widely known.^44,45^ The comparison of PCB chemical activity in fish with that in water through eq 1 provides valuable information (Figure 4). A major proportion of datapoints are <1 indicating a lower activity of PCBs in fish than in water. This means that despite these compounds being extremely lipophilic and assumed to biomagnify, levels in fish most often do not exceed the level of contamination in water. The range of ratios for fish from the sampling location in Oslofjord, Hvaler, and Ålesund sampled over a 4-year period is from 0.02 to 20.1. The median of these ratios for individual congeners ranges from 0.11 for CB180 at Hvaler to 0.52 for Cb153 at Ålesund. The median of ratios for CB28 at Jan Mayen and Andøya are similar and 0.41 (0.18–0.66) and 0.50 (0.48–2.0) but with a larger range of values for Andøya. This is the only congener for which the comparison was possible at these two sites. When SR data were below the LOQ, these were used to calculate the ratios, and these are given in Tables SI-9 and 10.

When the ratios are >1, these are equivalent to a TMF of maximum 1.5 to 2. Ratios are more consistently under 1 for CB28 and CB52 (in line with HCB) than those for other congeners. This is in line with our knowledge of biomagnification of PCBs and expected TMFs.^46,47^ One noticeable feature of Figure 4 is that ratios >1 are principally from Kristiansand. The median of activity ratios for CB28 and CB52 of 0.31 and 0.44 (range of 0.07 to 2.89), respectively, for the Kristiansand location are in line with those from other locations. For the remaining 5 PCB congeners, the median of activity ratios range from 1.0 (0.41–3.07) for CB101 to 3.57 (0.99–7.84) for CB153. The area, this sampling site is located in, is generally contaminated with organochlorinated compounds including PCBs. A mismatch in representativeness between the PS and fish monitoring is conceivable here^44^ since cod live deeper in water and may be exposed to higher concentrations close to the sediment–water interface in areas with PCB-contaminated sediment.^43^ This can be through water or through ingestion of contaminated sediment-dwelling preys. Except for the Kristiansand data, the data do not demonstrate a major increase in the contaminant level from water to fish. An explanation for the tendency for the thermodynamic activity ratios to be <1 was proposed by Smedes et al. (2020).^9^ At the base of the food chain, the thermodynamic activity of the contaminants in algae is not able to reach that in the water as a result of slow uptake and growth dilution. The metabolism/biotransformation of PCBs in fish (liver) may also play a role in the relative levels observed.^39^

**Figure 5:**
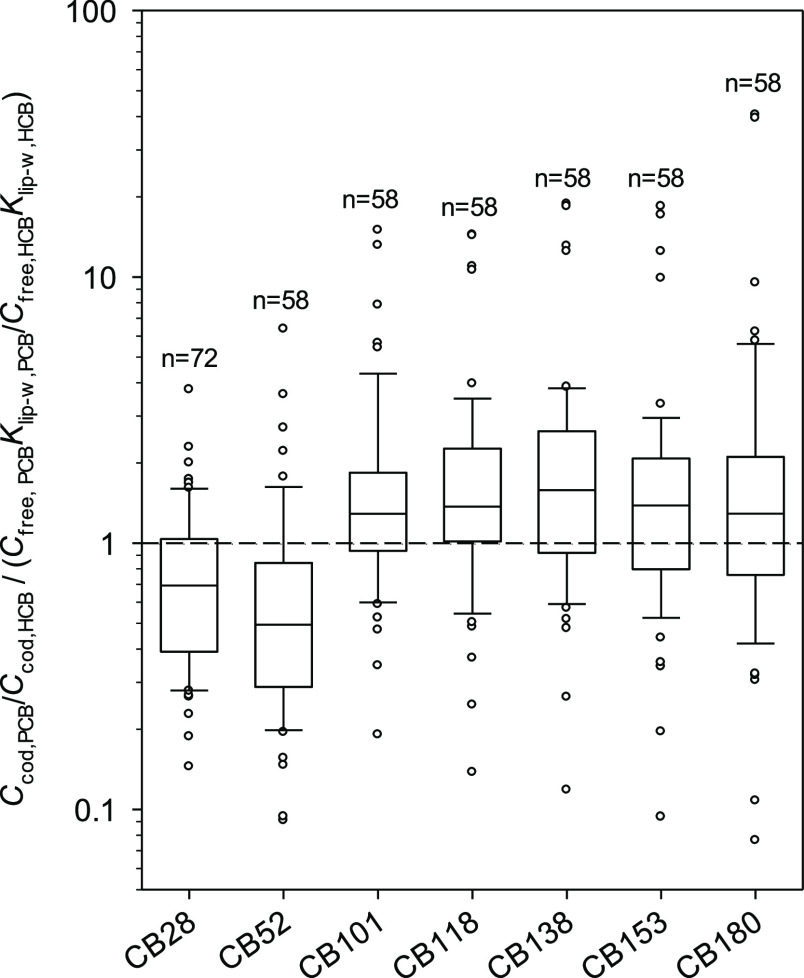
Benchmarking of PCB concentrations in *G. morhua* with those of HCB for fish from the Oslofjord, Andøya (CB28 only), and Jan Mayen (CB28 only). Ratios are further normalized to the ratio of products of *K*_lip-w,PCB_ and *C*_free,PCB_ over that for HCB. Note: a benchmarking ratio above 1 likely indicates biomagnification of that PCB congener relative to HCB.

A further benchmarking of PCB data with HCB assumed to represent steady-state bioconcentration in cod which combines eqs 2 and 3 is shown in Figure 5. These data are mostly for Oslofjord. Ratios >1 are likely for compounds undergoing biomagnification. For CB28 and CB52, ratios span from 0.09 to 6.4 with median values between 0.21 and 0.82 for the 4 years of monitoring in Oslofjord (2012–2016). The median of ratios for Jan Mayen for 2009 of 0.69 (0.36–1.6) is in line with the Oslofjord data. A higher median of ratios for Andøya (2010) of 1.46 (0.76–2.3) can be observed. In general, ratios below or close to 1 for CB28 and CB52 confirm that there is no major biomagnification of these two congeners when benchmarking with HCB. For CB101, CB118, CB138, CB153, and CB180, a much more significant proportion of data is above 1, indicating that these congeners are relatively more concentrated in cod liver than what could be expected from bioconcentration only. Ratios for these congeners range from 0.08 to 41 with median of ratios for each of the 4 years of monitoring spanning from 0.77 to 2.51. Overall, benchmarking with HCB reveals that the accumulation of PCBs in cod is not the result of lipid-water partitioning only. Processes involving biomagnification through diet are likely responsible for this relative increase in concentration of selected PCB congeners in cod.^33^

The interpretation of *C*_lip_ in fish using *K*_sr-w_ values and *K*_lip-sr_ was possible here. The use of *K*_lip-w_ is robust as a result of the low variability that has been observed for *K*_lip-sr_. These, however, differ from relatively more standard BCF measurements since they do not account for elimination processes and assume steady-state conditions. Previous attempts at benchmarking to help measure BCFs with musk xylene were justified by the authors because the chemical was not expected to be eliminated through metabolism to any significant extent, and it has a moderate BCF value in fish.^34^ Their data show that benchmarking with HCB results in a data correction similar to that achieved with musk xylene. Standardized BCF testing (OECD 305 guidelines) includes assessing the elimination kinetics after studying rates of uptake in a large number of fish. Because of the inherent variability involved in these procedures, the error associated with the BCF values obtained are relatively large and would make the comparisons in this study more difficult (particularly when these are based on whole body wet weight and not lipid-based) if based on such BCFs.

### Implications for Future Studies

The nature of the comparison of independently obtained contaminant concentrations observed in fish and the data theoretically-empirically deduced from PS reinforces our conclusions. HCB does not appear to biomagnify to any significant extent in freshwater or marine fish of Norway. Relative levels of HCB and PeCB in fish are mainly indicative of bioconcentration, or in the case of biomagnification of HCB, indicative of a TMF only slightly above 1. The implications of this are (i) the possibility to use not only PeCB for benchmarking bioconcentration but also HCB for which much more data exist and (ii) the incapacity to evidence biomagnification and reliable TMFs for HCB here that challenges the use of a proposed procedure for recalculating biota concentrations at a specific TL for comparison with WFD EQS set for that TL. For example, the TMF of 2.9 attributed by Fliedner et al. (2016) to HCB bioaccumulation in freshwater systems^48^ seems inappropriate. Our study also emphasizes that gathering biota data with simultaneous measurements of abiotic chemical activity in the surrounding environment is valuable.^9^ Ideally, this work could be improved by developing sets of paired fish-PS data at the national or EU level or through the aqua-GAPS/aqua-MONET network.^7,49,50^ These studies could also be extended to other potentially bioaccumulative chemicals. For this, robust values of *K*_sr-w_ and K_lip-sr_ are needed and can be obtained following published guidelines.^51^ Many studies have reported measurements of *K*_sr-w_ values^35,52,53^ while only a handful have reported *K*_lip-sr_.^13−15^ Further work should aim to fill in the gaps in the availability of these parameters to facilitate data interpretation for a wider range of chemicals.

Further information may be gained from looking at benchmarking with other organisms. For example, on average, HCB/PeCB ratios in three species of birds, Common Eider, European Shag, and Herring Gull from Norway, of 14.3, 25.1, and 20.6, respectively, do not deviate much from the ratio found in fish.^54^ Including other species such as cetaceans with a higher TL can sometime affect TMF estimates. The ratios for pilot whale liver from waters around the Faroe Islands were in the range of 25–39.^55^ Considering a generic TL of 4.4 for the pilot whale, this would be equivalent to a TMF of 1.5 relative to water which remains far from the proposed value of 2.9. Interestingly and for comparison, the HCB/PeCB ratio in explanted human silicone prostheses from Norway ranging from 9.7 to 93.8 with an average of 47.2 (sd = 21, *n* = 33) appears higher than in the aquatic species above.^56^

For the sites where the comparison was possible, PCB levels in cod were in general below those measured in the water these fish live in. This phenomenon has been shown before for a larger range of chemicals in freshwater fish.^9^ Despite indications of PCB biomagnification in cod, levels in fish struggle to reach those in water. Further work is needed to understand these processes. Considering the results presented here, PS measurements have a role to play in the monitoring of WFD priority substances for which EQS_biota_ have been derived.

## Supporting Information Available

Freshwater fish data for HCB and PeCB; cod data for HCB, PeCB, and PCBs from various coastal monitoring programs; PS-based freely dissolved concentrations, equilibrium concentrations in SR, and equivalent concentrations in lipids for PCBs, HCB, and PeCB at three sites on the Norwegian Coast; lipid-water partition coefficients for PeCB, HCB, and PCBs; and cod-water activity ratios for sampling locations where data from SR were below the LOQ (PDF)

## References

[ref1] Fliedner A.; Rüdel H.; Lohmann N.; Buchmeier G.; Koschorreck J. Biota monitoring under the Water Framework Directive: on tissue choice and fish species selection. Environ. Pollut. 2018, 235, 129–140. 10.1016/j.envpol.2017.12.052. 29276959

[ref2] Fliedner A.; Rüdel H.; Teubner D.; Buchmeier G.; Lowis J.; Heiss C.; Wellmitz J.; Koschorreck J. Biota monitoring and the Water Framework Directive-can normalization overcome shortcomings in sampling strategies?. Environ. Sci. Pollut. Res. 2016, 23, 21927–21939. 10.1007/s11356-016-7442-2. PMC5099357 27535154

[ref3] Moermond C. T.; Verbruggen E. M. An evaluation of bioaccumulation data for hexachlorobenzene to derive water quality standards according to the EU-WFD methodology. Integr. Environ. Assess. Manage. 2013, 9, 87–97. 10.1002/ieam.1351. 22791265

[ref4] Carere M.; Dulio V.; Hanke G.; Polesello S. Guidance for sediment and biota monitoring under the Common Implementation Strategy for the Water Framework Directive. TrAC, Trends Anal. Chem. 2012, 36, 15–24. 10.1016/j.trac.2012.03.005.

[ref5] Deutsch K.; Leroy D.; Belpaire C.; Den Haan K.; Vrana B.; Clayton H.; Hanke G.; Ricci M.; Held A.; Gawlik B. COMMON IMPLEMENTATION STRATEGY FOR THE WATER FRAMEWORK DIRECTIVE (2000/60/EC). Guidance Document No. 32 on Biota Monitoring (The Implementation of EQSBIOTA) under the Water Framework Directive; European Commission, 2013.

[ref6] Kidd K. A.; Burkhard L. P.; Babut M.; Borgå K.; Muir D. C.; Perceval O.; Ruedel H.; Woodburn K.; Embry M. R. Practical advice for selecting or determining trophic magnification factors for application under the European Union Water Framework Directive. Integr. Environ. Assess. Manage. 2019, 15, 266–277. 10.1002/ieam.4102. PMC6719707 30298984

[ref7] Booij K.; Robinson C. D.; Burgess R. M.; Mayer P.; Roberts C. A.; Ahrens L.; Allan I. J.; Brant J.; Jones L.; Kraus U. R.; Larsen M. M.; Lepom P.; Petersen J.; Pröfrock D.; Roose P.; Schäfer S.; Smedes F.; Tixier C.; Vorkamp K.; Whitehouse P. Passive sampling in regulatory chemical monitoring of nonpolar organic compounds in the aquatic environment. Environ. Sci. Technol. 2015, 50, 3–17. 10.1021/acs.est.5b04050. 26619247

[ref8] Gilbert D.; Witt G.; Smedes F.; Mayer P. Polymers as reference partitioning phase: polymer calibration for an analytically operational approach to quantify multimedia phase partitioning. Anal. Chem. 2016, 88, 5818–5826. 10.1021/acs.analchem.6b00393. 27115830

[ref9] Smedes F.; Sobotka J.; Rusina T. P.; Fialová P.; Carlsson P.; Kopp R.; Vrana B. Unraveling the Relationship between the Concentrations of Hydrophobic Organic Contaminants in Freshwater Fish of Different Trophic Levels and Water Using Passive Sampling. Environ. Sci. Technol. 2020, 54, 7942–7951. 10.1021/acs.est.9b07821. 32551598

[ref10] Mayer P.; Toräng L.; Glæsner N.; Jönsson J. Å. Silicone Membrane Equilibrator: Measuring Chemical Activity of Nonpolar Chemicals with Poly(dimethylsiloxane) Microtubes Immersed Directly in Tissue and Lipids. Anal. Chem. 2009, 81, 1536–1542. 10.1021/ac802261z. 19146462

[ref11] Allan I. J.; Bæk K.; Haugen T. O.; Hawley K. L.; Høgfeldt A. S.; Lillicrap A. D. In vivo passive sampling of nonpolar contaminants in brown trout (Salmo trutta). Environ. Sci. Technol. 2013, 47, 11660–11667. 10.1021/es401810r. 24020983

[ref12] Pintado-Herrera M. G.; Allan I. J.; González-Mazo E.; Lara-Martín P. A. Passive Samplers vs Sentinel Organisms: One-Year Monitoring of Priority and Emerging Contaminants in Coastal Waters. Environ. Sci. Technol. 2020, 54, 6693–6702. 10.1021/acs.est.0c00522. 32402185

[ref13] Jahnke A.; McLachlan M. S.; Mayer P. Equilibrium sampling: Partitioning of organochlorine compounds from lipids into polydimethylsiloxane. Chemosphere 2008, 73, 1575–1581. 10.1016/j.chemosphere.2008.09.017. 18926556

[ref14] Ruus A.; Allan I. J.; Bæk K.; Borgå K. Partitioning of persistent hydrophobic contaminants to different storage lipid classes. Chemosphere 2021, 263, 127890 10.1016/j.chemosphere.2020.127890. 32814130

[ref15] Smedes F.; Rusina T. P.; Beeltje H.; Mayer P. Partitioning of hydrophobic organic contaminants between polymer and lipids for two silicones and low density polyethylene. Chemosphere 2017, 186, 948–957. 10.1016/j.chemosphere.2017.08.044. 28830066

[ref16] Jahnke A.; MacLeod M.; Wickström H.; Mayer P. Equilibrium sampling to determine the thermodynamic potential for bioaccumulation of persistent organic pollutants from sediment. Environ. Sci. Technol. 2014, 48, 11352–11359. 10.1021/es503336w. 25184484

[ref17] Jahnke A.; Mayer P.; McLachlan M. S. Sensitive equilibrium sampling to study polychlorinated biphenyl disposition in Baltic Sea sediment. Environ. Sci. Technol. 2012, 46, 10114–10122. 10.1021/es302330v. 22916822

[ref18] Jahnke A.; Mayer P.; McLachlan M. S.; Wickström H.; Gilbert D.; MacLeod M. Silicone passive equilibrium samplers as ’chemometers’ in eels and sediments of a Swedish lake. Environmental Science: Processes & Impacts 2014, 16, 464–472. 10.1039/c3em00589e. 24448366

[ref19] Allan I. J.; Vrana B.; de Weert J.; Kringstad A.; Ruus A.; Christensen G.; Terentjev P.; Green N. W. Passive sampling and benchmarking to rank HOC levels in the aquatic environment. Sci. Rep. 2021, 11, 11231 10.1038/s41598-021-90457-3. 34045522 PMC8159932

[ref20] Allan I.; Jenssen M. T. S.; Braaten H. F. V. Priority Substances and Emerging Contaminants in Selected Norwegian Rivers–The River Monitoring Programme 2017; NIVA-rapport, 2018.

[ref21] Jartun M.; Økelsrud A.; Rundberget T.; Enge E. K.; Rostkowski P.; Warner N. A.; Harju M.; Johansen I. Monitoring of Environmental Contaminants in Freshwater Ecosystems 2018–Occurrence and Biomagnification; NIVA-rapport, 2019.

[ref22] Lyche J. L.; Nøstbakken O. J.; Berg V. EU Water Framework-Directive Priority Contaminants in Norwegian Freshwater Fish; EU, 2019.

[ref23] Green N.; Molvær J.; Kaste Ø.; Schrum C.; Yakushev E.; Sørensen K.; Allan I.; Høgåsen T.; Bjørkenes-Chr A. Tilførselsprogrammet 2009. Overvåkning av tilførsel og miljøtilstand i Barentshavet og Lofotenområdet; NIVA report, 2010; p 5980.

[ref24] Green N. W.; Heldal H. E.; Måge A.; Aas W.; Gäfvert T.; Schrum C.; Boitsov S.; Breivik K.; Iosjpe M.; Yakushev E. Tilførselsprogrammet 2010. Overvåking Av Tilførsler Og Miljøtilstand I Nordsjøen; NIVA, 2011.

[ref25] Green N. W.; Heldal H. E.; Måge A.; Aas W.; Gäfvert T.; Schrum C.; Boitsov S.; Breivik K.; Iosjpe M.; Yakushev E. Tilførselsprogrammet 2011. Overvåking Av Tilførsler Og Miljøtilstand I Norskehavet; NIVA, 2012.

[ref26] Green N.; Schøyen M.; Øxnevad S.; Ruus A.; Allan I.; Hjermann D.; Høgåsen T.; Beylich B.; Håvardstun J.; Lund E. Contaminants in coastal waters of Norway-2014. Miljøgifter I kystområdene 2014. Norwegian Environment Agency Miljødirektoratet, Monitoring report M-433| 2015; Norwegian Institute for Water Research project, 2015; Vol. 15330; p 6917.

[ref27] Green N.; Schøyen M.; Øxnevad S.; Ruus A.; Hjermann D.; Severinsen G.; Høgåsen T.; Beylich B.; Håvardstun J.; Lund E. Contaminants in coastal waters of Norway-2016. Miljøgifter I kystområdene 2015. Norwegian Environment Agency Miljødirektoratet, Monitoring report M-656| 2017; Norwegian Institute for Water Research project, 2017; Vol. 17330; p 7200.

[ref28] Green N. W.; Schøyen M.; Øxnevad S.; Ruus A.; Allan I.; Hjermann D. Ø.; Høgåsen T.; Beylich B.; Håvardstun J.; Lund E. Contaminants in Coastal Waters of Norway 2014; NIVA, 2015.

[ref29] Ruus A.; Bæk K.; Petersen K.; Allan I.; Beylich B.; Schlabach M.; Warner N.; Helberg M. Miljøgifter I en urban fjord, 2014. Environmental Contaminants In an Urban Fjord 2014; NIVA, 2015; p 6884.

[ref30] Schøyen M.; Allan I. J.; Ruus A.; Håvardstun J.; Hjermann D. Ø.; Beyer J. Comparison of caged and native blue mussels (Mytilus edulis spp.) for environmental monitoring of PAH, PCB and trace metals. Mar. Environ. Res. 2017, 130, 221–232. 10.1016/j.marenvres.2017.07.025. 28801106

[ref31] Green N. W.; Schøyen M.; Øxnevad S.; Ruus A.; Allan I.; Hjermann D.; Severinsen G.; Høgåsen T.; Beylich B.; Håvardstun J. Contaminants in Coastal Waters of Norway 2015. Miljøgifter I Norske Kystområder 2015; NIVA-rapport, 2016.

[ref32] Booij K.; Smedes F.; Crum S. Laboratory performance study for passive sampling of nonpolar chemicals in water. Environ. Toxicol. Chem. 2017, 36, 1156–1161. 10.1002/etc.3657. 27753131

[ref33] Kelly B. C.; Ikonomou M. G.; Blair J. D.; Morin A. E.; Gobas F. A. P. C. Food Web–Specific Biomagnification of Persistent Organic Pollutants. science 2007, 317, 236–239. 10.1126/science.1138275. 17626882

[ref34] Adolfsson-Erici M.; Åkerman G.; McLachlan M. S. Measuring bioconcentration factors in fish using exposure to multiple chemicals and internal benchmarking to correct for growth dilution. Environ. Toxicol. Chem. 2012, 31, 1853–1860. 10.1002/etc.1897. 22639194

[ref35] Smedes F. Silicone-water partition coefficients determined by cosolvent method for chlorinated pesticides, musks, organo phosphates, phthalates and more. Chemosphere 2018, 210, 662–671. 10.1016/j.chemosphere.2018.07.054. 30031996

[ref36] Inoue Y.; Hashizume N.; Yoshida T.; Murakami H.; Suzuki Y.; Koga Y.; Takeshige R.; Kikushima E.; Yakata N.; Otsuka M. Comparison of bioconcentration and biomagnification factors for poorly water-soluble chemicals using common carp (Cyprinus carpio L.). Arch. Environ. Contam. Toxicol. 2012, 63, 241–248. 10.1007/s00244-012-9761-8. 22484798

[ref37] Arnot J. A.; Gobas F. A. A review of bioconcentration factor (BCF) and bioaccumulation factor (BAF) assessments for organic chemicals in aquatic organisms. Environ. Rev. 2006, 14, 257–297. 10.1139/a06-005.

[ref38] Houde M.; Muir D. C. G.; Kidd K. A.; Guildford S.; Drouillard K.; Evans M. S.; Wang X.; Whittle D. M.; Haffner D.; Kling H. Influence of lake characteristics on the biomagnification of persistent organic pollutants in lake trout food webs. Environ. Toxicol. Chem. 2008, 27, 2169–2178. 10.1897/08-071.1. 18444699

[ref39] Ruus A.; Ugland K. I.; Skaare J. U. Influence of trophic position on organochlorine concentrations and compositional patterns in a marine food web. Environ. Toxicol. Chem. 2002, 21, 2356–2364. 10.1002/etc.5620211114. 12389914

[ref40] Burkhard L. P.; Borgå K.; Powell D. E.; Leonards P.; Muir D. C.; Parkerton T. F.; Woodburn K. B. Improving the Quality and Scientific Understanding of Trophic Magnification Factors (TMFs); ACS Publications, 2013. 10.1021/es305253r 23320844

[ref41] Økelsrud A.; Lydersen E.; Fjeld E. Biomagnification of mercury and selenium in two lakes in southern Norway. Sci. Total Environ. 2016, 566–567, 596–607. 10.1016/j.scitotenv.2016.05.109. 27236625

[ref42] Kim S.-K.; Kang C.-K. Temporal and spatial variations in hydrophobicity dependence of field-derived metrics to assess the biomagnification potential of hydrophobic organochlorine compounds. Sci. Total Environ. 2019, 690, 300–312. 10.1016/j.scitotenv.2019.06.221. 31295584

[ref43] Ruus A.; Daae I. A.; Hylland K. Accumulation of polychlorinated biphenyls from contaminated sediment by Atlantic cod (Gadus morhua): Direct accumulation from resuspended sediment and dietary accumulation via the polychaeteNereis virens. Environ. Toxicol. Chem. 2012, 31, 2472–2481. 10.1002/etc.1973. 22865726

[ref44] Kim J.; Gobas F. A. P. C.; Arnot J. A.; Powell D. E.; Seston R. M.; Woodburn K. B. Evaluating the roles of biotransformation, spatial concentration differences, organism home range, and field sampling design on trophic magnification factors. Sci. Total Environ. 2016, 551–552, 438–451. 10.1016/j.scitotenv.2016.02.013. 26891010

[ref45] Walters D. M.; Jardine T. D.; Cade B. S.; Kidd K. A.; Muir D. C. G.; Leipzig-Scott P. Trophic Magnification of Organic Chemicals: A Global Synthesis. Environ. Sci. Technol. 2016, 50, 4650–4658. 10.1021/acs.est.6b00201. 27014905

[ref46] Hallanger I. G.; Warner N. A.; Ruus A.; Evenset A.; Christensen G.; Herzke D.; Gabrielsen G. W.; Borgå K. Seasonality in contaminant accumulation in Arctic marine pelagic food webs using trophic magnification factor as a measure of bioaccumulation. Environ. Toxicol. Chem. 2011, 30, 1026–1035. 10.1002/etc.488. 21312250

[ref47] Kelly B. C.; Ikonomou M. G.; Blair J. D.; Gobas F. A. Bioaccumulation behaviour of polybrominated diphenyl ethers (PBDEs) in a Canadian Arctic marine food web. Sci. Total Environ. 2008, 401, 60–72. 10.1016/j.scitotenv.2008.03.045. 18538377

[ref48] Fliedner A.; Lohmann N.; Rüdel H.; Teubner D.; Wellmitz J.; Koschorreck J. Current levels and trends of selected EU Water Framework Directive priority substances in freshwater fish from the German environmental specimen bank. Environ. Pollut. 2016, 216, 866–876. 10.1016/j.envpol.2016.06.060. 27389550

[ref49] Miège C.; Mazzella N.; Allan I.; Dulio V.; Smedes F.; Tixier C.; Vermeirssen E.; Brant J.; O’Toole S.; Budzinski H.; Ghestem J.-P.; Staub P.-F.; Lardy-Fontan S.; Gonzalez J.-L.; Coquery M.; Vrana B. Position paper on passive sampling techniques for the monitoring of contaminants in the aquatic environment - Achievements to date and perspectives. Trends Environ. Anal. Chem. 2015, 8, 20–26. 10.1016/j.teac.2015.07.001.

[ref50] Lohmann R.; Muir D.; Zeng E. Y.; Bao L.-J.; Allan I. J.; Arinaitwe K.; Booij K.; Helm P.; Kaserzon S.; Mueller J. F. Aquatic Global Passive Sampling (AQUA-GAPS) Revisited: First Steps toward a Network of Networks for Monitoring Organic Contaminants in the Aquatic Environment; ACS Publications, 2017. 10.1021/acs.est.6b05159 27983810

[ref51] Booij K.; Smedes F.; Allan I. J. Guidelines for Determining Polymer-Water and Polymer-Polymer Partition Coefficients of Organic Compounds; ICES, 2017.

[ref52] Verhagen R.; O’Malley E.; Smedes F.; Mueller J. F.; Kaserzon S. Calibration parameters for the passive sampling of organic UV filters by silicone; diffusion coefficients and silicone-water partition coefficients. Chemosphere 2019, 223, 731–737. 10.1016/j.chemosphere.2019.02.077. 30807940

[ref53] Pintado-Herrera M. G.; Lara-Martín P. A.; González-Mazo E.; Allan I. J. Determination of silicone rubber and low-density polyethylene diffusion and polymer/water partition coefficients for emerging contaminants. Environ. Toxicol. Chem. 2016, 35, 2162–2172. 10.1002/etc.3390. 26833936

[ref54] Huber S.; Warner N. A.; Nygård T.; Remberger M.; Harju M.; Uggerud H. T.; Kaj L.; Hanssen L. A broad cocktail of environmental pollutants found in eggs of three seabird species from remote colonies in Norway. Environ. Toxicol. Chem. 2015, 34, 1296–1308. 10.1002/etc.2956. 25728907

[ref55] Hoydal K. S.; Letcher R. J.; Blair D. A. D.; Dam M.; Lockyer C.; Jenssen B. M. Legacy and emerging organic pollutants in liver and plasma of long-finned pilot whales (Globicephala melas) from waters surrounding the Faroe Islands. Sci. Total Environ. 2015, 520, 270–285. 10.1016/j.scitotenv.2015.03.056. 25817764

[ref56] Allan I. J.; Bæk K.; Kringstad A.; Roald H. E.; Thomas K. V. Should silicone prostheses be considered for specimen banking? A pilot study into their use for human biomonitoring. Environ. Int. 2013, 59, 462–468. 10.1016/j.envint.2013.06.021. 23955326

