# Supplementary material for: Passive Sampling Helps the Appraisal of Contaminant Bioaccumulation in Norwegian Fish Used for Regulatory Chemical Monitoring
Source: Environ Sci Technol. 2022 Jun 7;56(12):7945–53. doi: 10.1021/acs.est.2c00714 (PMC9228060; doi:10.1021/acs.est.2c00714)
Supplement: Supplementary file 1 — es2c00714_si_001.pdf [file es2c00714_si_001.pdf]

Passive sampling helps the appraisal of contaminant bioaccumulation in Norwegian fish used for regulatory chemical monitoring

IAN JOHN ALLAN [§]*, *BRANISLAV VRANA* [ξ] *and ANDERS RUUS* [§]

[§] *Norwegian Institute for Water Research, Økernveien 94, NO-0579, Oslo, Norway*

[ξ] *RECETOX, Faculty of Science, Masaryk University, Kotlarska 2, 61137 Brno, Czech Republic*

Number of pages: 24

Number of tables: 10

Number of figures: 0

Table SI-1	Fish data for HCB and PeCB (from the river monitoring programme)	P3
Table SI-2	Fish data for HCB and PeCB from the "Milfersk" monitoring programme.	P3
Table SI-3	Fish data for HCB and PeCB from Lyche et al. (2018)	P6
Table SI-4	Norwegian cod data for HCB, PeCB and PCBs from various monitoring programmes	P8
Table SI-5	Passive sampling-based freely dissolved concentrations of PCBs, HCB and PeCB at three sites on the Norwegian Coast (Oslofjord, Hvaler and Ålesund) monitoring from 2012 to 2016 (ng L^{-1}; grey shaded cells are for data below limits of quantification).	P17
Table SI-6	Calculated concentrations of PCBs, HCB and PeCB in silicone rubber (SR, AlteSil™) at equilibrium with the water at three sites on the Norwegian Coast (Oslofjord, Hvaler and Ålesund) monitoring from 2012 to 2016 (ng g^{-1}; grey shaded cells are for data below limits of quantification).	P20
Table SI-7	Equivalent concentrations of PCBs, HCB and PeCB in lipids at equilibrium with the water at three sites on the Norwegian Coast (Oslofjord, Hvaler and Ålesund) monitoring from 2012 to 2016 (ng g^{-1} lipid; grey shaded cells are for data below limits of quantification).	P21
Table SI-8	Lipid-water partition coefficients for pentachlorobenzene (PeCB), hexachlorobenzene (HCB) and PCBs.	P23
Table SI-9	Median cod-water activity ratios for sampling locations (Andøya, Bear Island and Jan Mayen) where data from the silicone rubber passive samplers (denominator in the activity ratio) were below limits of quantification.	P23

* Corresponding author: E-mail: ian.allan@niva.no. Tel. +47 22 18 5100, Fax. +47 22 18 5200

Table SI-10	Median cod-water activity ratios for sampling locations (Hvaler and Ålesund) where data from the silicone rubber passive samplers (denominator in the activity ratio) were below limits of quantification.	P23

METHODS

Freshwater biomonitoring data.

Table SI-1, 2 and 3 present concentrations of HCB and PeCB in freshwater fish from Norway as reported in the Norwegian environmental contaminants database (https://vannmiljo.miljodirektoratet.no/)

Marine biomonitoring data.

Cod biomonitoring data were obtained from the Norwegian environmental contaminants database (https://vannmiljo.miljodirektoratet.no/, accessed from 06-2019/06-2020) and/or monitoring reports by NIVA on behalf of the Norwegian Environment Agency. Table SI-4 present PeCB, HCB and PCB concentration in cod (liver) from the Norwegian coast.

Passive sampling data

Freely dissolved concentrations, silicone rubber concentrations at equilibrium and equivalent concentrations in lipids at equilibrium with the water, calculated from passive sampling for exposure from 2012 to 2016 in the Oslofjord, at Hvaler and in Ålesund are reported in Tables SI-5, 6 and 7. Freely dissolved concentrations were calculated from mass of contaminants accumulated in the silicone rubber samplers and from the performance reference compound dissipation (PRC) data. Polymer-water partition coefficients for HCB, PeCB, PCBs and PRCs were from Smedes et al. (2009)[1]. The non-linear least square method was applied to the PRC data to estimate silicone-water exchange kinetics[2] and contaminant sampling rates were estimated using the model from Rusina et al., (2009)[3]. This methodology has been used over the past decade[4, 5].

Table SI-1. Fish data for HCB and PeCB (from the river monitoring programme)

River ID	Species	Date	Sample type (number of fish/fillet pooled)	lipid % (EOM)	Concentration (ng g^{-1} wet weight) HCB	PeCH
Alna	Salmo trutta	2018	Pooled whole fish (x5)	3.85	1.10	0.086
Alna	Salmo trutta	2018	Pooled whole fish (x11)	3.86	1.23	0.12
Alna	Salmo trutta	2018	Pooled fillet (x4)	3.42	1.25	0.18
Gaula	Salmo salar	2018	Pooled whole fish (x5)	3.69	0.38	0.022
Gaula	Salmo salar	2018	Pooled whole fish (x5)	4.22	0.47	0.026
Gaula	Salmo salar	2018	Pooled whole fish (x5)	4.48	0.46	0.028
Ørsta	Salmo salar	2018	Pooled whole fish (x5)	4.46	0.29	0.019
Ørsta	Salmo salar	2018	Pooled whole fish (x5)	2.21	0.18	< 0.01
Ørsta	Salmo salar	2018	Pooled whole fish (x4)	2.19	0.19	0.012
Nausta	Salmo trutta	2018	Pooled whole fish (x5)	2.35	0.23	0.036
Nausta	Salmo trutta	2018	Pooled whole fish (x3)	2.31	0.21	0.025
Nausta	Salmo trutta	2018	Pooled liver/fillet (x2)	1.12	0.096	< 0.01
Ranaelva	Salmo salar	2018	Pooled whole fish (x6)	2.15	0.61	< 0.01
Ranaelva	Salmo salar	2018	Pooled whole fish (x6)	1.53	0.30	0.014
Ranaelva	Salmo salar	2018	Pooled whole fish (x6)	1.75	0.36	0.018

Table SI-2. Fish data for HCB and PeCB from the "Milfersk" monitoring programme.

Location code	Lake ID	Species	Date	Sample type	lipid %	Concentration (ng g^{-1} wet weight) HCB	PeCH
196-38195	Altevatnet (Álddesjávri)	Salvelinus alpinus	2018-08-28	Liver	3.26	0.372	0.014
196-38195	Altevatnet (Álddesjávri)	Salvelinus alpinus	2018-08-28	Liver	3.75	0.408	0.014
196-38195	Altevatnet (Álddesjávri)	Salvelinus alpinus	2018-08-28	Liver	2.54	0.271	0.011
043-29689	Bergesvatnet	Salmo trutta	2018-08-22	Liver	3.03	0.016	0.018
043-29689	Bergesvatnet	Salmo trutta	2018-08-22	Liver	2.52	0.094	0.01
043-29689	Bergesvatnet	Salmo trutta	2018-08-22	Liver	3.27	0.015	0.018
021-43368	Byglandsfjorden	Salmo trutta	2017-09-15	Liver	2.81	0.241	0.015
021-43368	Byglandsfjorden	Salmo trutta	2017-09-15	Liver	3.23	0.272	0.017
021-43368	Byglandsfjorden	Salmo trutta	2017-09-15	Liver	2.87	0.272	0.014
104-87246	Eikesdalsvatnet	Salmo trutta	2017-08-22	Liver	3.52	0.506	0.027

104-87246	Eikesdalsvatnet	Salmo trutta	2017-08-22	Liver	2.63	0.282	0.019
104-87246	Eikesdalsvatnet	Salmo trutta	2017-08-22	Liver	4.27	0.947	0.045
311-82851	Femunden	Salmo trutta	2015-09-01	Fillet	0.73	0.233	0.018
311-82851	Femunden	Salmo trutta	2015-09-01	Fillet	0.03	0.132	0.013
311-82851	Femunden	Salmo trutta	2015-09-01	Fillet	0.42	0.002	0.011
311-82851	Femunden	Salmo trutta	2015-09-01	Fillet	1.47	0.675	0.027
311-82851	Femunden	Salmo trutta	2015-09-01	Fillet	1.34	0.027	0.023
311-82851	Femunden	Salmo trutta	2015-09-01	Fillet	0.26	0.169	0.009
311-82851	Femunden	Salmo trutta	2015-09-01	Fillet	2	0.477	0.025
311-82851	Femunden	Salmo trutta	2015-09-01	Fillet	0.52	0.023	0.013
311-82851	Femunden	Salmo trutta	2015-09-01	Fillet	0.05	0.135	0.009
311-82851	Femunden	Salmo trutta	2015-09-01	Fillet	1.37	0.415	0.026
311-82851	Femunden	Salmo trutta	2015-09-01	Fillet	0.91	0.248	0.01
311-82851	Femunden	Salmo trutta	2015-09-01	Fillet	1.01	0.476	0.022
311-82851	Femunden	Salmo trutta	2015-09-0	Fillet	0.84	0.271	0.015
311-82851	Femunden	Salmo trutta	2015-09-0	Fillet	0.64	0.212	0.014
311-82851	Femunden	Salmo trutta	2015-09-01	Fillet	0.72	0.214	0.015
194-84806	Geitvatnet	Salmo trutta	2018-09-06	Liver	3.16	0.348	0.02
194-84806	Geitvatnet	Salmo trutta	2018-09-06	Liver	3.58	0.295	0.013
194-84806	Geitvatnet	Salvelinus alpinus	2018-09-06	Liver	11.01	0.997	0.056
089-38147	Hornindalsvatnet	Salmo trutta	2017-11-19	Liver	2.54	0.154	0.009
089-38147	Hornindalsvatnet	Salmo trutta	2017-11-19	Liver	4.18	0.303	0.012
089-38147	Hornindalsvatnet	Salmo trutta	2017-11-19	Liver	4.35	0.286	0.018
234-37977	Iešjávri	Salvelinus alpinus	2018-08-20	Liver	1.92	0.243	0.012
234-37977	Iešjávri	Salvelinus alpinus	2018-08-20	Liver	2.08	0.293	0.011
234-37977	Iešjávri	Salvelinus alpinus	2018-08-20	Liver	4.55	0.501	0.021
137-91154	Kangsvatnet	Salmo trutta	2018-10-03	Liver	2.93	0.448	0.016
137-91154	Kangsvatnet	Salmo trutta	2018-10-03	Liver	3.04	0.307	0.014
137-91154	Kangsvatnet	Salmo trutta	2018-10-03	Liver	3.55	0.353	0.015
012-43517	Krøderen	Perca fluviatilis	2018-09-17	Liver	2.03	0.132	0.007
012-43517	Krøderen	Perca fluviatilis	2018-09-17	Liver	3.08	0.229	0.01
012-43517	Krøderen	Perca fluviatilis	2018-09-17	Liver	3.48	0.232	0.013
139-91162	Langvatnet	Salmo trutta	2018-10-03	Liver	3.05	0.302	0.015
026-38724	Lundevatnet	Salmo trutta	2017-08-15	Liver	4.27	0.179	0.011
026-38724	Lundevatnet	Salmo trutta	2017-08-15	Liver	5.45	0.206	0.013
026-38724	Lundevatnet	Salmo trutta	2017-08-15	Liver	5.28	0.023	0.013
002-37942	Lyseren	Salmo trutta	2018-09-07	Liver	2.34	0.126	0.005
002-37942	Lyseren	Perca fluviatilis	2018-09-07	Liver	2.31	0.139	0.007
002-37942	Lyseren	Perca fluviatilis	2018-09-07	Liver	2.22	0.011	0.007
002-86866	Mingevannet	Perca fluviatilis	2017-11-19	Liver	3.13	0.021	0.012
002-44093	Mjøsa (F)	Salmo trutta	2015-09-01	Fillet	2.41	0.454	0.029
002-44093	Mjøsa (F)	Salmo trutta	2015-09-01	Fillet	3	0.653	0.036
002-44093	Mjøsa (F)	Salmo trutta	2015-09-01	Fillet	3.02	0.068	0.046
002-44093	Mjøsa (F)	Salmo trutta	2015-09-01	Fillet	9.09	1.082	0.125
002-44093	Mjøsa (F)	Salmo trutta	2015-09-01	Fillet	2.04	0.532	0.035
002-44093	Mjøsa (F)	Salmo trutta	2015-09-01	Fillet	5.77	1.001	0.075
002-44093	Mjøsa (F)	Salmo trutta	2015-09-01	Fillet	2.96	0.593	0.037

002-44093	Mjøsa (F)	Salmo trutta	2015-09-01	Fillet	6.28	1.001	0.065
002-44093	Mjøsa (F)	Salmo trutta	2015-09-01	Fillet	4.45	0.095	0.061
002-44093	Mjøsa (F)	Salmo trutta	2015-09-01	Fillet	2.82	1	0.057
002-44093	Mjøsa (F)	Salmo trutta	2015-09-01	Fillet	1.56	0.278	0.021
002-44093	Mjøsa (F)	Salmo trutta	2015-09-01	Fillet	1.37	0.305	0.019
002-44093	Mjøsa (F)	Salmo trutta	2015-09-01	Fillet	0.78	0.257	0.017
002-44093	Mjøsa (F)	Salmo trutta	2015-09-01	Fillet	4.63	1.001	0.071
002-44093	Mjøsa (F)	Salmo trutta	2015-09-01	Fillet	1.78	0.408	0.023
016-38185	Møsvatn	Salvelinus alpinus	2018-10-05	Liver	7.02	1.036	0.038
016-38185	Møsvatn	Salvelinus alpinus	2018-10-05	Liver	4.37	0.515	0.019
016-38185	Møsvatn	Salvelinus alpinus	2018-10-05	Liver	4.32	0.499	0.021
012-82852	Randsfjorden	Salmo trutta	2015-09-0	Fillet	0.85	0.216	0.015
012-82852	Randsfjorden	Salmo trutta	2015-09-01	Fillet	0.95	0.234	0.017
012-82852	Randsfjorden	Salmo trutta	2015-09-01	Fillet	0.78	0.206	0.015
012-82852	Randsfjorden	Salmo trutta	2015-09-01	Fillet	1.22	0.257	0.016
012-82852	Randsfjorden	Salmo trutta	2015-09-01	Fillet	3	0.664	0.048
012-82852	Randsfjorden	Salmo trutta	2015-09-01	Fillet	2.28	0.551	0.039
012-82852	Randsfjorden	Salmo trutta	2015-09-01	Fillet	2.04	0.488	0.036
012-82852	Randsfjorden	Salmo trutta	2015-09-01	Fillet	0.52	0.194	0.017
012-82852	Randsfjorden	Salmo trutta	2015-09-01	Fillet	3.11	0.796	0.049
012-82852	Randsfjorden	Salmo trutta	2015-09-01	Fillet	10.05	1.058	0.093
012-82852	Randsfjorden	Salvelinus alpinus	2015-09-01	Fillet	0.67	0.496	0.02
012-82852	Randsfjorden	Salvelinus alpinus	2015-09-01	Fillet	1.86	0.316	0.024
012-82852	Randsfjorden	Salvelinus alpinus	2015-09-01	Fillet	0.34	0.199	0.013
012-82852	Randsfjorden	Salvelinus alpinus	2015-09-01	Fillet	1.71	0.048	0.025
012-82852	Randsfjorden	Salvelinus alpinus	2015-09-01	Fillet	0.79	0.222	0.013
002-38084	Selsvatnet	Salmo trutta	2017-07-20	Whole fish	5.82	0.131	0.02
002-38084	Selsvatnet	Salmo trutta	2017-07-20	Whole fish	5.66	0.156	0.023
002-38084	Selsvatnet	Salmo trutta	2017-07-20	Liver	3.07	0.006	0.008
234-89853	Smalfjordvannet	Salvelinus alpinus	2017-08-08	Liver	2.76	0.287	0.012
234-89853	Smalfjordvannet	Salmo trutta	2017-08-08	Liver	1.05	0.098	< 0.005
234-89853	Smalfjordvannet	Salmo trutta	2017-08-08	Liver	2.01	0.168	0.008
177-84819	Steinvatnet	Salmo trutta	2018-09-08	Liver	2.88	0.546	0.022
177-84819	Steinvatnet	Salmo trutta	2018-09-08	Liver	2.68	0.032	0.014
177-84819	Steinvatnet	Salvelinus alpinus	2018-09-08	Liver	3.18	0.027	0.016
313-80085	Storbørja	Perca fluviatilis	2018-09-07	Liver	3.72	0.197	0.01
313-80085	Storbørja	Perca fluviatilis	2018-09-07	Liver	3.17	0.165	0.009
313-80085	Storbørja	Perca fluviatilis	2018-09-07	Liver	3.63	0.164	0.009
231-89857	Storvannet (Gamvik)	Salvelinus alpinus	2017-08-07	Liver	3.34	0.446	0.024
231-89857	Storvannet (Gamvik)	Salmo trutta	2017-08-07	Liver	4.03	0.536	0.021
231-89857	Storvannet (Gamvik)	Salmo trutta	2017-08-07	Liver	3.91	0.468	0.021
212-80664	Stuorajávri	Perca fluviatilis	2018-08-22	Liver	6.05	0.401	0.018
212-80664	Stuorajávri	Perca fluviatilis	2018-08-22	Liver	5.55	0.355	0.02
002-87244	Surtningen	Salmo trutta	2017-08-15	Liver	3.39	0.016	0.01
002-87244	Surtningen	Salmo trutta	2017-08-15	Liver	3.28	0.159	0.012
002-87244	Surtningen	Salmo trutta	2017-08-15	Liver	3.76	0.227	0.014
012-82853	Tyrifjorden	Perca fluviatilis	2015-09-01	Fillet	0.24	0.289	0.045

012-82853	Tyrifjorden	Perca fluviatilis	2015-09-01	Fillet	1.26	0.057		0.017
012-82853	Tyrifjorden	Perca fluviatilis	2015-09-01	Fillet	1.57	0.048		0.016
012-82853	Tyrifjorden	Perca fluviatilis	2015-09-01	Fillet	0.68	0.084		0.014
012-82853	Tyrifjorden	Perca fluviatilis	2015-09-01	Fillet	2.87	0.115		0.017
062-43286	Vangsvatnet	Salmo trutta	2017-08-08	Liver	1.96	0.148		0.006
062-43286	Vangsvatnet	Salmo trutta	2017-08-08	Liver	3.51	0.252		0.01
062-43286	Vangsvatnet	Salvelinus alpinus	2017-08-08	Liver	3.21	0.568		0.02
003-82854	Vansjø	Perca fluviatilis	2015-09-01	Fillet	0.14	0.034	<	0.005
003-82854	Vansjø	Perca fluviatilis	2015-09-01	Fillet	0.03	0.036		0.009
003-82854	Vansjø	Perca fluviatilis	2015-09-01	Fillet	0.18	0.033	<	0.005
003-82854	Vansjø	Perca fluviatilis	2015-09-01	Fillet	0.16	0.039		0.01
003-82854	Vansjø	Perca fluviatilis	2015-09-01	Fillet	0.25	0.052		0.008
009-89875	Øvre Drengsrudvann	Perca fluviatilis	2017-09-21	Liver	3.03	0.203		0.016
009-89875	Øvre Drengsrudvann	Perca fluviatilis	2017-09-21	Liver	3.74	0.025		0.023
009-89875	Øvre Drengsrudvann	Perca fluviatilis	2017-09-21	Liver	3.62	0.223		0.02
002-61186	Øyeren ved Bjørnstad	Perca fluviatilis	2017-08-31	Liver	4.66	0.384		0.022
002-61186	Øyeren ved Bjørnstad	Perca fluviatilis	2017-08-31	Liver	3.35	0.293		0.017
002-61186	Øyeren ved Bjørnstad	Perca fluviatilis	2017-08-31	Liver	3.47	0.317		0.018
084-82146	Åsvatnet	Salmo trutta	2017-08-31	Liver	2.05	0.278		0.018

Table SI-3. Fish data for HCB and PeCB from Lyche et al. (2018)[6]

						Concentration (ng g^{-1} wet weight)	
Location code	Lake ID	Species	Date	Sample type	lipid %	HCB	PeCH
	Lyseren	Salmo trutta	summer/autumn 2018	Pooled liver	2.34	0.126	0.005
	Lyseren	Perca Fluviatilis	summer/autumn 2019	Pooled liver	2.31	0.139	0.007
	Lyseren	Perca Fluviatilis	summer/autumn 2020	Pooled liver	2.22	0.111	0.007
	Storbørja	Perca Fluviatilis	summer/autumn 2021	Pooled liver	3.72	0.197	0.01
	Storbørja	Perca Fluviatilis	summer/autumn 2022	Pooled liver	3.17	0.165	0.009
	Storbørja	Perca Fluviatilis	summer/autumn 2023	Pooled liver	3.63	0.164	0.009
	Bergervatnet	Salmo trutta	summer/autumn 2024	Pooled liver	3.3	0.16	0.018
	Bergervatnet	Salmo trutta	summer/autumn 2025	Pooled liver	2.52	0.094	0.01
	Bergervatnet	Salmo trutta	summer/autumn 2026	Pooled liver	3.27	0.15	0.018
	Kangsvatnet	Salmo trutta	summer/autumn 2027	Pooled liver	2.93	0.448	0.016
	Kangsvatnet	Salmo trutta	summer/autumn 2028	Pooled liver	3.4	0.307	0.014
	Kangsvatnet	Salmo trutta	summer/autumn 2029	Pooled liver	3.55	0.353	0.015
	Langevann	Salmo trutta	summer/autumn 2030	Pooled liver	3.5	0.302	0.015
	Steinvatn	Salmo trutta	summer/autumn 2031	Pooled liver	2.88	0.546	0.022
	Steinvatn	Salmo trutta	summer/autumn 2032	Pooled liver	2.68	0.32	0.015
	Steinvatn	Salmo trutta	summer/autumn 2033	Pooled liver	3.18	0.27	0.016
	Geitvatnet	Salmo trutta	summer/autumn 2034	Pooled liver	3.16	0.348	0.02
	Geitvatnet	Salmo trutta	summer/autumn 2035	Pooled liver	3.58	0.295	0.013
	Geitvatnet	Salvelinus alpinus	summer/autumn 2036	Pooled liver	11.11	0.997	0.056
	lesjavri	Salvelinus alpinus	summer/autumn 2037	Pooled liver	1.92	0.243	0.012

lesjavri	Salvelinus alpinus	summer/autumn 2038	Pooled liver	2.8	0.293	0.011
lesjavri	Salvelinus alpinus	summer/autumn 2039	Pooled liver	4.55	0.501	0.021
stuorajarvri	Perca Fluviatilis	summer/autumn 2040	Pooled liver	6.05	0.401	0.018
stuorajarvri	Perca Fluviatilis	summer/autumn 2041	Pooled liver	5.55	0.355	0.02
Altevannbardu	Salvelinus alpinus	summer/autumn 2042	Pooled liver	3.26	0.372	0.014
Altevannbardu	Salvelinus alpinus	summer/autumn 2043	Pooled liver	3.75	0.408	0.014
Altevannbardu	Salvelinus alpinus	summer/autumn 2044	Pooled liver	2.54	0.271	0.011
Møsvatn	Salvelinus alpinus	summer/autumn 2045	Pooled liver	7.02	1.036	0.038
Møsvatn	Salvelinus alpinus	summer/autumn 2046	Pooled liver	4.37	0.515	0.019
Møsvatn	Salvelinus alpinus	summer/autumn 2047	Pooled liver	4.32	0.499	0.021
vaggatem	white fish	summer/autumn 2048	Pooled liver	3.11	0.232	0.01
vaggatem	white fish	summer/autumn 2049	Pooled liver	3.39	0.164	0.007
vaggatem	white fish	summer/autumn 2050	Pooled liver	3.74	0.24	0.013
tangenfossen	white fish	summer/autumn 2051	Pooled liver	3.51	0.265	0.015
tangenfossen	white fish	summer/autumn 2052	Pooled liver	2.28	0.298	0.011
tangenfossen	white fish	summer/autumn 2053	Pooled liver	2.68	0.234	0.01
Krøderen	Perca Fluviatilis	summer/autumn 2054	Pooled liver	2.03	0.132	0.007
Krøderen	Perca Fluviatilis	summer/autumn 2055	Pooled liver	3.08	0.229	0.01
Krøderen	Perca Fluviatilis	summer/autumn 2056	Pooled liver	3.48	0.232	0.013

Table SI-4. Norwegian cod data for HCB, PeCB and PCBs from various monitoring programmes

(Grey shaded cells are for data below limits of quantification)

Location code	site ID	Species	Date	Sample type	lipid %	Concentration ($ng\ g^{-1}$ wet weight) HCB	PeCH	CB28	CB52	CB101	CB118	CB138	CB153	CB180
	Andøya	Gadus morhua	2009	Liver	56	7.2	1.2	2	2.3	5.9	7.1	11	13	2.5
	Andøya	Gadus morhua	2009	Liver	63	5.5	1.5	2	2	5.3	4.8	7.6	9.9	2
	Andøya	Gadus morhua	2009	Liver	65	4.2	1	2	2	3.3	3.6	5.7	7.7	2
	Andøya	Gadus morhua	2009	Liver	62	5.9	1.5	2	2.3	5.9	4.9	7.8	9.9	2
	Andøya	Gadus morhua	2009	Liver	54	5	1	2	2.5	5.4	5.5	7.6	9.6	2
	Andøya	Gadus morhua	2009	Liver	62	5.3	1.2	2	2.3	5.7	5.3	7.8	9.5	2
	Andøya	Gadus morhua	2009	Liver	69	5.5	1.5	3	3	4.5	3.5	5.5	6.2	3
	Andøya	Gadus morhua	2009	Liver	64	11	2.3	2	5.2	16	22	32	55	10
	Andøya	Gadus morhua	2009	Liver	41	15	2	5.2	12	29	38	50	68	13
	Andøya	Gadus morhua	2009	Liver	49	8.4	2	2	5.1	11	11	16	23	4.1
	Andøya	Gadus morhua	2009	Liver	67	10	1.7	2.1	4.7	10	10	14	19	4.1
	Svalbard	Gadus morhua	2009	Liver	55	7.5	1.1	1	1.2	5.1	5.3	4.7	6.9	1.2
	Svalbard	Gadus morhua	2009	Liver	40	8.8	0.88	1	1.9	7	7.3	5.9	9.3	2.1
	Svalbard	Gadus morhua	2009	Liver	48	15	1.5	1.6	3.7	8.5	13	9.5	12	2.5
	Svalbard	Gadus morhua	2009	Liver	54	12	1.4	1.2	2.3	6.2	8.4	5	7.2	1.2
	Svalbard	Gadus morhua	2009	Liver	48	8	0.89	1	2.2	4.3	7	6.1	8.8	1.8
	Svalbard	Gadus morhua	2009	Liver	49	11	1.2	1.1	3.8	11	13	12	16	3.3
	Svalbard	Gadus morhua	2009	Liver	45	6.5	0.94	1	1.3	3.1	5.1	5.1	7.8	1.6
	Svalbard	Gadus morhua	2009	Liver	58	8.5	1.3	1	1.7	4.2	5.8	4.1	5.6	1
	Svalbard	Gadus morhua	2009	Liver	32	7.8	0.88	1	1.9	6.5	6.7	6.9	10	2.5
	Svalbard	Gadus morhua	2009	Liver	51	8.6	1	1	2	4.5	5.9	4.1	6.2	1
	Svalbard	Gadus morhua	2009	Liver	50	7.7	1.2	1	1.8	3.8	5.6	4.7	6.4	1.3
	Svalbard	Gadus morhua	2009	Liver	52	12	1.3	2.1	3.8	7.2	9.3	6.1	8.6	1.7
	Svalbard	Gadus morhua	2009	Liver	62	8.7	1.1	1	2.6	3.9	5.9	3.9	6.3	1.4
	Svalbard	Gadus morhua	2009	Liver	47	10	1.2	1	2.9	7.6	7.7	5.9	7.5	1.5
	Svalbard	Gadus morhua	2009	Liver	49	4.8	0.99	1	1.6	3.8	3.5	2.6	3.6	1
	Svalbard	Gadus morhua	2009	Liver	54	9	1.2	1	3	4	7.3	4.9	6.8	1.4

Svalbard	Gadus morhua	2009	Liver	46	14	1.2	1.7	6	13	17	15	21	5.3
Svalbard	Gadus morhua	2009	Liver	40	6	0.72	1	2.1	4.6	5.2	5.5	7.5	1.7
Svalbard	Gadus morhua	2009	Liver	58	10	1.2	1	2.7	5.7	7.4	5.2	7	1.4
Svalbard	Gadus morhua	2009	Liver	54	7.3	0.95	1	2.3	6.3	6	4.6	6.3	1.1
Svalbard	Gadus morhua	2009	Liver	53	7.9	1.6	1	2	6	5.8	5	6.6	1.1
Svalbard	Gadus morhua	2009	Liver	43	7.6	1	1	1.8	3.5	5.4	4.5	6.6	1.7
Svalbard	Gadus morhua	2009	Liver	43	7.9	0.98	1	3.4	6.5	8	5.6	7.4	1.5
Svalbard	Gadus morhua	2009	Liver	60	6.9	1	1	1.4	6.5	3.7	2	2.9	1
Svalbard	Gadus morhua	2009	Liver	56	6.8	1.1	1	1.7	4.1	5.5	3.5	4.7	1
JanMayen	Gadus morhua	2011	Liver	55	28	1.6	2.9	13	26	39	52	61	15
JanMayen	Gadus morhua	2011	Liver	69	37	1.6	4.6	20	31	30	47	51	12
JanMayen	Gadus morhua	2011	Liver	64	31	1.7	3.4	15	30	37	45	49	12
JanMayen	Gadus morhua	2011	Liver	49	22	1.2	3.4	12	19	36	53	55	15
JanMayen	Gadus morhua	2011	Liver	53	31	1.9	3.4	13	21	25	35	39	9
JanMayen	Gadus morhua	2011	Liver	59	17	1.2	1.4	6.4	10	15	20	24	5.6
JanMayen	Gadus morhua	2011	Liver	62	21	1.6	1.8	7.1	14	21	26	33	8.2
JanMayen	Gadus morhua	2011	Liver	62	28	2.1	2.7	10	19	26	36	40	9.8
JanMayen	Gadus morhua	2011	Liver	69	25	2.1	6	17	24	31	43	46	11
JanMayen	Gadus morhua	2011	Liver	38	18	2.1	3.3	13	19	22	36	42	10
JanMayen	Gadus morhua	2011	Liver	63	30	2.1	1.6	8	19	25	35	40	10
JanMayen	Gadus morhua	2011	Liver	58	30	2.1	3.1	10	15	19	32	37	10
JanMayen	Gadus morhua	2011	Liver	61	34	2	3.3	11	16	16	27	28	6.4
JanMayen	Gadus morhua	2011	Liver	58	35	2.5	3.2	9.8	12	13	19	21	4.7
JanMayen	Gadus morhua	2011	Liver	55	28	1.7	2.9	10	15	18	27	30	7.4
JanMayen	Gadus morhua	2011	Liver	59	29	2	2.8	9.6	13	15	24	26	6.2
JanMayen	Gadus morhua	2011	Liver	51	30	2	3.1	11	15	17	27	29	6.8
JanMayen	Gadus morhua	2011	Liver	57	29	1.6	4.6	12	17	22	37	39	11
JanMayen	Gadus morhua	2011	Liver	55	37	2.2	3.7	12	18	20	33	37	9.4
Kvænangen	Gadus morhua	2009	Liver	32	5.5	2.6	1	2.5	11	16	24	44	7.7
Kvænangen	Gadus morhua	2009	Liver	11	2.9	0.57	1.1	2.8	10	17	25	37	7.1
Kvænangen	Gadus morhua	2009	Liver	43	5.8	1.4	1	3.4	13	19	32	60	9.9
Kvænangen	Gadus morhua	2009	Liver	39	5.9	1.9	1	2.5	7.4	9.4	15	27	5.7
Kvænangen	Gadus morhua	2009	Liver	67	8.9	2.7	1.4	4.6	13	15	22	35	6.5

Kvænangen	Gadus morhua	2009	Liver	43	7.9	2	1.5	5.9	21	32	59	84	19
Kvænangen	Gadus morhua	2009	Liver	46	18	3.7	3.3	11	26	34	44	63	13
Kvænangen	Gadus morhua	2009	Liver	30	9.8	1.6	2.6	7.9	28	37	55	85	17
Kvænangen	Gadus morhua	2009	Liver	60	13	2.9	1.9	6.2	13	19	26	41	8.3
Kvænangen	Gadus morhua	2009	Liver	59	14	2.6	1.9	6.4	19	24	33	50	10
Kvænangen	Gadus morhua	2009	Liver	41	13	2	2.1	6.5	12	15	17	26	5.2
Kvænangen	Gadus morhua	2009	Liver	56	9.3	2.7	1.5	4.3	16	20	30	49	9.7
Kvænangen	Gadus morhua	2009	Liver	42	6.6	2.1	1	2.7	9.4	14	20	35	6.1
Kvænangen	Gadus morhua	2009	Liver	36	5.2	1.1	1	3.1	16	22	38	60	11
Kvænangen	Gadus morhua	2009	Liver	34	4.8	1.2	1	1.7	7.2	11	17	34	5.7
Kvænangen	Gadus morhua	2009	Liver	44	10	2	1.9	5.9	20	26	41	63	13
Kvænangen	Gadus morhua	2009	Liver	49	8.4	2	1.3	4.7	15	18	27	46	8.8
Kvænangen	Gadus morhua	2009	Liver	45	7.6	2.1	1.2	4.2	13	17	24	40	7.9
Kvænangen	Gadus morhua	2009	Liver	37	6.6	2.2	1	3.4	13	22	41	65	14
Kvænangen	Gadus morhua	2009	Liver	24	11	1.7	2.4	17	52	53	74	120	22
Kvænangen	Gadus morhua	2009	Liver	55	8.6	2.1	1.5	5.3	15	18	27	41	8.1
Egersundbanken	Gadus morhua	2010	Liver	25.3	5.6	1	0.99	2.4	11	13	36	49	6.5
Egersundbanken	Gadus morhua	2010	Liver	55.6	10	1	1.9	3.6	12	13	31	41	5.7
Egersundbanken	Gadus morhua	2010	Liver	47.9	12	1.3	2.2	5.5	21	21	49	64	10
Egersundbanken	Gadus morhua	2010	Liver	56.2	8.2	1.6	2.1	2.6	7.9	11	24	32	3.3
Egersundbanken	Gadus morhua	2010	Liver	56.2	8.2	1.6	2.1	2.6	7.9	11	24	32	3.3
Egersundbanken	Gadus morhua	2010	Liver	24.3	4.5	4.1	1.3	1.7	5.4	8.7	17	22	2.1
Egersundbanken	Gadus morhua	2010	Liver	24.3	2.4	1	0.41	0.19	2.4	5	10	15	2
Egersundbanken	Gadus morhua	2010	Liver	24.3	7.5	1.1	2.8	4.6	15	15	36	45	6.7
Egersundbanken	Gadus morhua	2010	Liver	40.3	9.4	1.5	3.4	6.3	17	19	43	56	7.7
Egersundbanken	Gadus morhua	2010	Liver	61	9.5	1.8	2.2	2.9	8.6	9.6	21	27	3.3
Egersundbanken	Gadus morhua	2010	Liver	62.7	15	1.6	2.8	4.8	15	15	33	43	6
Egersundbanken	Gadus morhua	2010	Liver	34.7	8.7	0.85	2.5	5.2	15	12	29	36	5
Egersundbanken	Gadus morhua	2010	Liver	25.3	7.3	3.1	1.3	2.7	11	13	30	40	5.2
Egersundbanken	Gadus morhua	2010	Liver	47.6	13	1.5	3.3	6.3	22	19	46	57	8.6
Egersundbanken	Gadus morhua	2010	Liver	36.2	8.5	1	2.9	5	17	16	36	41	6.1
Egersundbanken	Gadus morhua	2010	Liver	31.1	7.2	1.5	1.9	4.9	23	25	70	87	13
Egersundbanken	Gadus morhua	2010	Liver	27.6	3.9	1	1.1	1.8	9.9	13	35	47	7.3

Egersundbanken	Gadus morhua	2010	Liver	46.8	2.7	1	1.2	1.6	7.8	8.8	22	31	5
Egersundbanken	Gadus morhua	2010	Liver	46.8	6.8	1	1.4	2.7	11	18	49	69	14
Egersundbanken	Gadus morhua	2010	Liver	46.8	9.2	1.2	1.1	0.4	2.4	12	27	51	9.1
Egersundbanken	Gadus morhua	2010	Liver	33.2	7.9	1.5	1.3	1.6	7.8	26	66	110	22
Egersundbanken	Gadus morhua	2010	Liver	7	2.7	1	1.4	1.9	10	12	36	54	7.3
Egersundbanken	Gadus morhua	2010	Liver	28.7	11	1.5	3.8	9.8	33	31	96	140	26
Egersundbanken	Gadus morhua	2010	Liver	37.1	10	1.1	2.2	3.4	11	13	29	41	6.3
Egersundbanken	Gadus morhua	2010	Liver	55.2	9.3	2	2.2	3.3	8.4	9.4	19	23	2.2
Egersundbanken	Gadus morhua	2010	Liver	47.6	1.7	0.9	0.62	1.3	9.8	13	33	48	7.1
Egersundbanken	Gadus morhua	2010	Liver	50.1	14	1.5	3.5	6.8	22	21	49	60	8.8
Bear Island	Gadus morhua	2009	Liver	60.8	18	2	3.8	8.8	13	9.8	14	14	2.4
Bear Island	Gadus morhua	2009	Liver	62.1	31	1.6	4.8	8.9	11	13	15	15	2.3
Bear Island	Gadus morhua	2009	Liver	58.5	18	1.5	3	6	11	11	15	17	3.3
Bear Island	Gadus morhua	2009	Liver	53.7	17	1.5	3.7	9.4	18	16	23	24	4.2
Bear Island	Gadus morhua	2009	Liver	64.8	22	1.5	3.1	6.6	11	9.5	13	15	2.9
Bear Island	Gadus morhua	2009	Liver	64.6	13	1.5	2	3.2	3.5	2.8	3.7	3.7	0.65
Bear Island	Gadus morhua	2009	Liver	28.9	27	1.6	5.3	14	27	28	43	50	11
Bear Island	Gadus morhua	2009	Liver	57.8	26	1.5	4.2	7.8	8.8	9.9	12	14	2.6
Bear Island	Gadus morhua	2009	Liver	45.4	26	1.5	6.7	14	28	35	42	51	9.4
Bear Island	Gadus morhua	2009	Liver	55.6	16	1.6	2.3	4.4	6.2	7.3	10	11	2
Bear Island	Gadus morhua	2009	Liver	45.8	24	1.5	2.7	4.8	9.4	12	16	22	4.5
Bear Island	Gadus morhua	2009	Liver	62.1	19	2.1	2	3.4	4	4.1	5.8	6.4	1.4
Bear Island	Gadus morhua	2009	Liver	60.3	19	1.9	4	3.7	5	4.2	5.3	6	1
Bear Island	Gadus morhua	2009	Liver	60.3	18	1.5	4	6.8	10	9.1	12	13	2.1
Bear Island	Gadus morhua	2009	Liver	50.2	11	1.5	2.2	2	3.9	3.6	4.6	5.5	1
Bear Island	Gadus morhua	2009	Liver	65.2	18	2	3.6	5.7	7.9	9.4	12	11	1.9
Bear Island	Gadus morhua	2009	Liver	61.3	18	2	2.7	3.9	4.9	5.8	8.3	8.1	1.7
Bear Island	Gadus morhua	2009	Liver	50.6	30	2.7	3.7	4.8	5.9	4.2	5.3	5.6	0.88
Bear Island	Gadus morhua	2009	Liver	57.4	16	1.7	3.7	3.3	5	4.8	7	7.7	1.5
Bear Island	Gadus morhua	2009	Liver	57.6	16	2.7	4.1	4.6	5.6	4.9	6.8	7	1.4
Bear Island	Gadus morhua	2009	Liver	44.5	12	1.5	2.3	3.1	5.9	7.2	9.1	11	2.1
Bear Island	Gadus morhua	2009	Liver	40.9	12	1.5	2.5	2.8	3.6	4.3	4.8	6.5	1.2
Bear Island	Gadus morhua	2009	Liver	55.8	25	2.4	4.5	5.7	5.3	4.8	5.8	6.1	1

Bear Island	Gadus morhua	2009	Liver	58.2	16	1.7	4.3	5.4	6.2	7.5	9	8.6	1.6
Bear Island	Gadus morhua	2009	Liver	56.3	15	1.5	3.5	3.9	4.2	4.2	4.9	5	0.82
Kristiansandsfjorden, Kongsgårdbukta	Gadus morhua	2010-08-01	Liver	34	43.7	1.46	9.4	30.7	184	258	283	334	489
Kristiansandsfjorden, Vesterhavn	Gadus morhua	2010-08-01	Liver	10	19.1	4.65	2.74	14.7	19.8	38.6	106	275	139
Kristiansandsfjorden, Vesterhavn	Gadus morhua	2010-08-01	Liver	16	11.3	4.63	1.04	3.79	55.9	55.2	101.8	175	369
Kristiansandsfjorden, Vesterhavn	Gadus morhua	2010-08-01	Liver	11	99.8	2.06	1.71	10.3	28.6	60.7	159	182	85
Kristiansandsfjorden, Vestergapet	Gadus morhua	2010-08-01	Liver	17	4.55	0.73	3.5	5.36	17.6	36.2	84.6	197	45.9
Kristiansandsfjorden, Kongsgårdbukta	Gadus morhua	2012-10-01	Liver	44	11.94	0.88	3.38	9.3	44.44	92.7	658	197.6	183.4
Kristiansandsfjorden, Vesterhavn	Gadus morhua	2012-10-01	Liver	18	34.56	1.28	2.85	3.73	103	175	429	562	61.4
Kristiansandsfjorden, Vesterhavn	Gadus morhua	2012-10-01	Liver	48.8	42.86	0.95	1.73	4.11	26.5	48.25	207	112	308
Kristiansandsfjorden, Vesterhavn	Gadus morhua	2012-10-01	Liver	37	13.81	4.62	4.68	6.96	31.1	110.8	208	298	140.7
Kristiansandsfjorden, Vestergapet	Gadus morhua	2012-10-01	Liver	44	11.03	0.56	0.72	2.23	21.7	38.6	126	151	72.6
Kristiansandsfjorden, Kongsgårdbukta	Gadus morhua	2010-08-01	Fillet	0.38	0.03	0.01							
Kristiansandsfjorden, Vesterhavn	Gadus morhua	2010-08-01	Fillet	0.37	0.32	0.02							
Kristiansandsfjorden, Vesterhavn	Gadus morhua	2010-08-01	Fillet	0.38	0.41	0.04							
Kristiansandsfjorden, Vesterhavn	Gadus morhua	2010-08-01	Fillet	0.41	0.14	0.03							
Kristiansandsfjorden, Vestergapet	Gadus morhua	2010-08-01	Fillet	0.39	0.03	0.01							
Kristiansandsfjorden, Kongsgårdbukta	Gadus morhua	2012-10-01	Fillet	0.42	0.09074	0.01							
Kristiansandsfjorden, Vesterhavn	Gadus morhua	2012-10-01	Fillet	0.44	0.36504	0.02713							
Kristiansandsfjorden, Vesterhavn	Gadus morhua	2012-10-01	Fillet	0.45	0.27455	0.02228							
Kristiansandsfjorden, Vesterhavn	Gadus morhua	2012-10-01	Fillet	0.43	0.24746	0.02016							
Kristiansandsfjorden, Vestergapet	Gadus morhua	2012-10-01	Fillet	0.51	0.10608	0.01							
Kristiansand havn (13B)	Gadus morhua	2015	Liver	21.5			4.6	5.3	25	110	220	390	180
Kristiansand havn (13B)	Gadus morhua	2015	Liver	30.6			43	12	58	150	310	470	210
Kristiansand havn (13B)	Gadus morhua	2015	Liver	21.3			6.7	5.2	17	49	120	210	100
Kristiansand havn (13B)	Gadus morhua	2015	Liver	8.3			15	19	15	57	120	250	110
Kristiansand havn (13B)	Gadus morhua	2015	Liver	51.7			61	35	52	150	270	450	210
Kristiansand havn (13B)	Gadus morhua	2015	Liver	39.6			16	13	25	45	100	150	55
Kristiansand havn (13B)	Gadus morhua	2015	Liver	31.6			2.4	2.9	14	64	150	290	130
Kristiansand havn (13B)	Gadus morhua	2015	Liver	32.6			3.4	6.3	25	59	180	390	220
Kristiansand havn (13B)	Gadus morhua	2015	Liver	57.9			72	27	46	89	170	240	92
Kristiansand havn (13B)	Gadus morhua	2015	Liver	17.1			12	12	57	180	270	450	200
Kristiansand havn (13B)	Gadus morhua	2015	Liver	24.6			3.9	5.3	32	66	210	470	280
Kristiansand havn (13B)	Gadus morhua	2015	Liver	31.4			4.2	5.6	28	77	190	380	200
Kristiansand havn (13B)	Gadus morhua	2015	Liver	24.3			16	13	54	130	280	420	190
Kristiansand havn (13B)	Gadus morhua	2015	Liver	23.9			3.7	8.9	45	130	230	450	230
Kristiansand havn (13B)	Gadus morhua	2016	Liver	31			3.4	3.5	15	28	78	150	60

Kristiansand havn (13B)	Gadus morhua	2016	Liver	52	2.9	4.4	24	58	170	560	300
Kristiansand havn (13B)	Gadus morhua	2016	Liver	38	11	6.4	30	61	150	250	120
Kristiansand havn (13B)	Gadus morhua	2016	Liver	36	1.6	3	11	23	57	81	27
Kristiansand havn (13B)	Gadus morhua	2016	Liver	50	2.8	4.8	17	49	110	170	65
Kristiansand havn (13B)	Gadus morhua	2016	Liver	31	6	5.6	18	100	240	470	240
Kristiansand havn (13B)	Gadus morhua	2016	Liver	43	2.3	3.2	9.3	16	33	44	11
Kristiansand havn (13B)	Gadus morhua	2016	Liver	56	2.9	5.2	15	19	47	70	26
Kristiansand havn (13B)	Gadus morhua	2016	Liver	42	2	3.5	11	19	44	68	19
Kristiansand havn (13B)	Gadus morhua	2016	Liver	53	3.5	5	18	62	120	230	83
Kristiansand havn (13B)	Gadus morhua	2016	Liver	54	2.4	5.2	19	39	57	77	16
Kristiansand havn (13B)	Gadus morhua	2016	Liver	64	2.7	4.4	15	21	50	73	21
Kristiansand havn (13B)	Gadus morhua	2016	Liver	52	4.8	2.8	5.6	29	69	120	35
Kristiansand havn (13B)	Gadus morhua	2016	Liver	41	2.8	4.1	24	53	150	220	70
Hvalerbassenget, Kirkøy nord (02B)	Gadus morhua	2012-10-01	Liver	36.6		13	39	83	220	280	69
Hvalerbassenget, Kirkøy nord (02B)	Gadus morhua	2012-10-01	Liver	24.5		2.1	9	23	61	100	18
Hvalerbassenget, Kirkøy nord (02B)	Gadus morhua	2012-10-01	Liver	35.2		17	37	59	130	150	37
Hvalerbassenget, Kirkøy nord (02B)	Gadus morhua	2012-10-01	Liver	26.9		13	43	100	270	340	90
Hvalerbassenget, Kirkøy nord (02B)	Gadus morhua	2012-10-01	Liver	12.7		8.5	24	61	140	190	47
Hvalerbassenget, Kirkøy nord (02B)	Gadus morhua	2012-10-01	Liver	17.5		6.9	23	65	150	200	48
Hvalerbassenget, Kirkøy nord (02B)	Gadus morhua	2013-10-05	Liver	25.2		3.1	6.7	48	67	130	34
Hvalerbassenget, Kirkøy nord (02B)	Gadus morhua	2013-10-05	Liver	19.4		6	19	45	76	150	34
Hvalerbassenget, Kirkøy nord (02B)	Gadus morhua	2013-10-05	Liver	32.6		5.6	11	25	44	72	18
Hvalerbassenget, Kirkøy nord (02B)	Gadus morhua	2013-10-05	Liver	43.8		7	12	28	49	77	19
Hvalerbassenget, Kirkøy nord (02B)	Gadus morhua	2014-10-03	Liver	35.1	2	5.4	16	42	76	150	35
Hvalerbassenget, Kirkøy nord (02B)	Gadus morhua	2014-10-03	Liver	35.7	3.9	5.7	19	52	110	170	33
Hvalerbassenget, Kirkøy nord (02B)	Gadus morhua	2014-10-03	Liver	5.9	1	1	1	9.1	20	36	8.9
Hvalerbassenget, Kirkøy nord (02B)	Gadus morhua	2015-09-13	Liver	22.6	41	62	24	56	68	120	44
Hvalerbassenget, Kirkøy nord (02B)	Gadus morhua	2015-09-13	Liver	34	1.6	4.1	5.4	17	34	56	17
Hvalerbassenget, Kirkøy nord (02B)	Gadus morhua	2015-09-13	Liver	30	2.6	5.4	12	41	75	120	31
Hvalerbassenget, Kirkøy nord (02B)	Gadus morhua	2015-09-13	Liver	6.6	1.2	2.2	2.4	11	25	51	15
Hvalerbassenget, Kirkøy nord (02B)	Gadus morhua	2015-09-13	Liver	15.6	7.3	15	9.2	47	60	120	34
Hvalerbassenget, Kirkøy nord (02B)	Gadus morhua	2016-09-05	Liver	33	2.5	6.8	14	32	49	75	23
Hvalerbassenget, Kirkøy nord (02B)	Gadus morhua	2016-09-0	Liver	38	1.6	2.5	9	23	48	68	15
Hvalerbassenget, Kirkøy nord (02B)	Gadus morhua	2016-09-05	Liver	13	1	1	4.6	16	31	55	16
Hvalerbassenget, Kirkøy nord (02B)	Gadus morhua	2016-09-05	Liver	27	2.4	5.6	16	59	96	150	38
Hvalerbassenget, Kirkøy nord (02B)	Gadus morhua	2016-09-05	Liver	18	1.3	1.9	10	27	64	92	20
Oslo-området, Indre Oslofjord (30B)	Gadus morhua	2012-11-05	Liver	49.4		93	190	380	860	1200	330
Oslo-området, Indre Oslofjord (30B)	Gadus morhua	2012-11-05	Liver	28.9		44	190	430	82	1500	420

Oslo-området, Indre Oslofjord (30B)	Gadus morhua	2012-11-05	Liver	54.1				58	190	570	1200	1600	400
Oslo-området, Indre Oslofjord (30B)	Gadus morhua	2012-11-05	Liver	34.2				27	78	240	430	570	150
Oslo-området, Indre Oslofjord (30B)	Gadus morhua	2012-11-05	Liver	10.5				18	90	240	640	920	280
Oslo-området, Indre Oslofjord (30B)	Gadus morhua	2012-11-05	Liver	38.8				34	85	410	860	1200	320
Oslo-området, Indre Oslofjord (30B)	Gadus morhua	2012-11-05	Liver	41.3				49	200	560	1300	1700	520
Oslo-området, Indre Oslofjord (30B)	Gadus morhua	2012-11-05	Liver	47.7				260	930	1500	3400	3600	870
Oslo-området, Indre Oslofjord (30B)	Gadus morhua	2012-11-05	Liver	47.9				58	190	420	1100	1300	350
Oslo-området, Indre Oslofjord (30B)	Gadus morhua	2012-11-05	Liver	37.4				57	290	690	1600	2100	580
Oslo-området, Indre Oslofjord (30B)	Gadus morhua	2012-11-05	Liver	28.2				41	130	300	640	950	250
Oslo-området, Indre Oslofjord (30B)	Gadus morhua	2012-11-05	Liver	29.7				34	48	250	410	690	230
Oslo-området, Indre Oslofjord (30B)	Gadus morhua	2012-11-05	Liver	62.3				110	310	550	1200	1400	360
Oslo-området, Indre Oslofjord (30B)	Gadus morhua	2012-11-05	Liver	5.6				3.7	25	100	310	420	160
Oslo-området, Indre Oslofjord (30B)	Gadus morhua	2012-11-05	Liver	42.4				95	510	1000	2400	2900	740
Oslo-området, Indre Oslofjord (30B)	Gadus morhua	2013-11-04	Liver	34.6	5.1	0.44		22	60	360	810	1400	390
Oslo-området, Indre Oslofjord (30B)	Gadus morhua	2013-11-04	Liver	36.2	14	0.59		280	890	1500	3100	4300	1300
Oslo-området, Indre Oslofjord (30B)	Gadus morhua	2013-11-04	Liver	31.5	5.1	0.49		53	170	630	1100	1800	460
Oslo-området, Indre Oslofjord (30B)	Gadus morhua	2013-11-04	Liver	74.7	15	0.96		110	330	570	1200	1700	470
Oslo-området, Indre Oslofjord (30B)	Gadus morhua	2013-11-04	Liver	58.2	6.3	0.6		24	87	240	670	1200	380
Oslo-området, Indre Oslofjord (30B)	Gadus morhua	2013-11-04	Liver	10.3	1.1	0.3		12	130	490	1200	2000	700
Oslo-området, Indre Oslofjord (30B)	Gadus morhua	2013-11-04	Liver	74.6	11	0.87		68	140	210	500	660	180
Oslo-området, Indre Oslofjord (30B)	Gadus morhua	2013-11-04	Liver	81.1	23	1.09		200	370	410	690	870	200
Oslo-området, Indre Oslofjord (30B)	Gadus morhua	2013-11-04	Liver	53	9	0.63		54	140	370	850	1400	390
Oslo-området, Indre Oslofjord (30B)	Gadus morhua	2013-11-04	Liver	72.8	11	0.92		44	130	280	600	950	280
Oslo-området, Indre Oslofjord (30B)	Gadus morhua	2013-11-04	Liver	30.1	3.2	0.33		21	79	160	390	620	180
Oslo-området, Indre Oslofjord (30B)	Gadus morhua	2013-11-04	Liver	65.4	13	0.77		110	260	410	960	1500	430
Oslo-området, Indre Oslofjord (30B)	Gadus morhua	2013-11-04	Liver	71	14	0.91		110	300	590	1100	1700	420
Oslo-området, Indre Oslofjord (30B)	Gadus morhua	2013-11-04	Liver	6.9	0.87	0.3		8.5	71	390	930	1700	570
Oslo-området, Indre Oslofjord (30B)	Gadus morhua	2013-11-04	Liver	44.5	6.2	0.55		22	55	160	310	470	110
Oslo-området, Indre Oslofjord (30B)	Gadus morhua	2013-11-04	Liver	54.8	7.6	0.89		31	60	130	310	490	120
Oslo-området, Indre Oslofjord (30B)	Gadus morhua	2014-11-05	Liver	63	10	0.72	14	89	220	380	620	800	160
Oslo-området, Indre Oslofjord (30B)	Gadus morhua	2014-11-05	Liver	53.3	5.4	0.58	5.6	38	140	240	600	850	200
Oslo-området, Indre Oslofjord (30B)	Gadus morhua	2014-11-05	Liver	32.5	6.4	1	3.6	16	80	260	700	1000	280
Oslo-området, Indre Oslofjord (30B)	Gadus morhua	2014-11-05	Liver	48.7	5.8	0.63	6.1	34	120	220	530	750	200

Oslo-området, Indre Oslofjord (30B)	Gadus morhua	2014-11-05	Liver	61.7	6.1	1.2	10	45	100	150	320	410	100
Oslo-området, Indre Oslofjord (30B)	Gadus morhua	2014-11-05	Liver	46.1	5	0.45	4.4	29	110	200	430	630	130
Oslo-området, Indre Oslofjord (30B)	Gadus morhua	2014-11-05	Liver	46.5	6	0.62	6.9	38	130	270	570	830	170
Oslo-området, Indre Oslofjord (30B)	Gadus morhua	2014-11-05	Liver	43.1	5.3	0.36	6.6	31	120	220	480	690	150
Oslo-området, Indre Oslofjord (30B)	Gadus morhua	2014-11-05	Liver	31.9	5.3	0.75	7.2	29	140	330	770	1200	280
Oslo-området, Indre Oslofjord (30B)	Gadus morhua	2014-11-05	Liver	43.9	6.2	0.38	6.9	34	110	230	490	720	150
Oslo-området, Indre Oslofjord (30B)	Gadus morhua	2014-11-05	Liver	40	12	0.63	4.8	31	130	290	580	850	190
Oslo-området, Indre Oslofjord (30B)	Gadus morhua	2014-11-05	Liver	40.6	7.7	0.43	6.4	31	110	250	500	730	150
Oslo-området, Indre Oslofjord (30B)	Gadus morhua	2014-11-05	Liver	37.8	6.3	0.49	6.4	27	110	240	500	730	170
Oslo-området, Indre Oslofjord (30B)	Gadus morhua	2014-11-05	Liver	53.3	3.6	0.49	6.3	29	90	180	440	660	170
Oslo-området, Indre Oslofjord (30B)	Gadus morhua	2014-11-05	Liver	40.4	5.9	0.6	8.3	51	160	350	940	1500	440
Oslo-området, Indre Oslofjord (30B)	Gadus morhua	2015-11-02	Liver	6.8	0.68	0.3	1	4	19	93	230	360	110
Oslo-området, Indre Oslofjord (30B)	Gadus morhua	2015-11-02	Liver	22.2	2.2	0.3	2.2	18	98	250	620	830	250
Oslo-området, Indre Oslofjord (30B)	Gadus morhua	2015-11-02	Liver	25.1	3.2	0.3	3	28	130	310	730	990	310
Oslo-området, Indre Oslofjord (30B)	Gadus morhua	2015-11-02	Liver	24.6	2.3	0.3	1.9	17	65	180	380	560	150
Oslo-området, Indre Oslofjord (30B)	Gadus morhua	2015-11-02	Liver	56.2	27	3.3	22	140	110	150	280	460	110
Oslo-området, Indre Oslofjord (30B)	Gadus morhua	2015-11-02	Liver	30.2	3.1	0.3	2.4	20	81	240	550	800	210
Oslo-området, Indre Oslofjord (30B)	Gadus morhua	2015-11-02	Liver	21.3	2.1	0.3	2.4	11	46	190	480	720	200
Oslo-området, Indre Oslofjord (30B)	Gadus morhua	2015-11-02	Liver	13.4	1.8	0.3	1.9	17	75	200	480	710	210
Oslo-området, Indre Oslofjord (30B)	Gadus morhua	2015-11-02	Liver	22.5	2.5	0.3	2.8	20	80	230	490	680	190
Oslo-området, Indre Oslofjord (30B)	Gadus morhua	2015-11-0	Liver	21.4	2.5	0.3	2.6	17	68	220	530	800	230
Oslo-området, Indre Oslofjord (30B)	Gadus morhua	2015-11-02	Liver	20.2	2.1	0.3	2	15	69	260	520	800	240
Oslo-området, Indre Oslofjord (30B)	Gadus morhua	2015-11-02	Liver	47.3	6.4	0.3	7.5	37	92	130	320	400	110
Oslo-området, Indre Oslofjord (30B)	Gadus morhua	2016-11-07	Liver	28	8.8	1	8.4	49	350	910	2200	3000	920
Oslo-området, Indre Oslofjord (30B)	Gadus morhua	2016-11-07	Liver	71	14	1.1	17	140	380	480	870	930	270
Oslo-området, Indre Oslofjord (30B)	Gadus morhua	2016-11-08	Liver	63	11	1	12	79	210	260	490	530	120
Oslo-området, Indre Oslofjord (30B)	Gadus morhua	2016-11-07	Liver	31	13	1	20	170	790	1600	3300	4600	1300
Oslo-området, Indre Oslofjord (30B)	Gadus morhua	2016-11-07	Liver	65	11	1	12	92	210	230	390	400	87
Oslo-området, Indre Oslofjord (30B)	Gadus morhua	2016-11-07	Liver	65	8.1	1	8.7	60	180	310	630	730	170
Oslo-området, Indre Oslofjord (30B)	Gadus morhua	2016-11-07	Liver	63	9.4	1	11	63	130	130	240	260	53
Oslo-området, Indre Oslofjord (30B)	Gadus morhua	2016-11-07	Liver	62	7.8	1	8.2	49	180	370	880	1100	290
Oslo-området, Indre Oslofjord (30B)	Gadus morhua	2016-11-07	Liver	52	8.1	1	11	75	200	320	500	570	140
Oslo-området, Indre Oslofjord (30B)	Gadus morhua	2016-11-08	Liver	62	10	1	8	50	130	230	480	610	150

Oslo-området, Indre Oslofjord (30B)	Gadus morhua	2016-11-07	Liver	55	6.8	1	8.1	40	130	270	640	890	250
Oslo-området, Indre Oslofjord (30B)	Gadus morhua	2016-11-07	Liver	56	7.8	1	7.4	38	50	130	290	390	100
Oslo-området, Indre Oslofjord (30B)	Gadus morhua	2016-11-07	Liver	35	2.9	1	4	22	68	170	380	520	160
Oslo-området, Indre Oslofjord (30B)	Gadus morhua	2016-11-07	Liver	48	5.3	1	6.1	36	120	320	800	1100	300
Oslo-området, Indre Oslofjord (30B)	Gadus morhua	2016-11-07	Liver	43	4.9	1	4.2	25	110	290	780	1100	330
Ålesund, område ved Hundsvær (28B)	Gadus morhua	2012-10-01	Liver	2.5				1	1	5.5	8.8	20	7.7
Ålesund, område ved Hundsvær (28B)	Gadus morhua	2012-10-01	Liver	24.6				4.8	12	81	130	250	110
Ålesund, område ved Hundsvær (28B)	Gadus morhua	2012-10-01	Liver	60.2				8.8	24	81	160	280	120
Ålesund, område ved Hundsvær (28B)	Gadus morhua	2012-10-01	Liver	9.5				2.1	19	100	240	410	200
Ålesund, område ved Hundsvær (28B)	Gadus morhua	2012-10-01	Liver	46.7				3.7	14	94	280	540	380
Ålesund, område ved Hundsvær (28B)	Gadus morhua	2012-10-01	Liver	6.4				1.7	11	36	93	170	76
Ålesund, område ved Hundsvær (28B)	Gadus morhua	2013-10-05	Liver	38.7				8.1	49	160	390	690	360
Ålesund, område ved Hundsvær (28B)	Gadus morhua	2013-10-05	Liver	8.1				7.3	27	87	200	270	110
Ålesund, område ved Hundsvær (28B)	Gadus morhua	2013-10-05	Liver	52.6				2.7	7	8.7	21	34	12
Ålesund, område ved Hundsvær (28B)	Gadus morhua	2013-10-05	Liver	39.5				100	340	600	1300	1800	600
Ålesund, område ved Hundsvær (28B)	Gadus morhua	2014-10-03	Liver	34.8			16	52	51	120	340	520	290
Ålesund, område ved Hundsvær (28B)	Gadus morhua	2014-10-03	Liver	38.7			110	140	69	86	140	170	95
Ålesund, område ved Hundsvær (28B)	Gadus morhua	2014-10-03	Liver	33.1			3.7	12	5.6	8.1	17	25	8.1
Ålesund, område ved Hundsvær (28B)	Gadus morhua	2015-09-13	Liver	41.4			130	190	84	130	240	370	200
Ålesund, område ved Hundsvær (28B)	Gadus morhua	2015-09-13	Liver	57.8			10	33	20	28	62	99	42
Ålesund, område ved Hundsvær (28B)	Gadus morhua	2015-09-13	Liver	33.7			26	130	170	330	820	1200	610
Ålesund, område ved Hundsvær (28B)	Gadus morhua	2015-09-13	Liver	48			1.2	3.8	8.9	11	25	29	7.1
Ålesund, område ved Hundsvær (28B)	Gadus morhua	2015-09-13	Liver	48			1.8	2.2	9.3	53	97	180	61
Ålesund, område ved Hundsvær (28B)	Gadus morhua	2016-09-05	Liver	41			1	2.4	7.2	21	61	77	31
Ålesund, område ved Hundsvær (28B)	Gadus morhua	2016-09-05	Liver	40			1	1	2.5	17	30	71	26
Ålesund, område ved Hundsvær (28B)	Gadus morhua	2016-09-05	Liver	55			4	7.7	28	63	120	170	43
Ålesund, område ved Hundsvær (28B)	Gadus morhua	2016-09-05	Liver	31			4.1	5.7	14	25	44	62	15
Indre Oslofjord, Midtmeie	Gadus morhua	2015-08-20	Liver	33.2	4.73	0.508							
Indre Oslofjord, Midtmeie	Gadus morhua	2015-08-20	Liver	41.1	4.4	0.546							
Indre Oslofjord, Midtmeie	Gadus morhua	2015-08-20	Liver	70.8	13.8	1.26							
Indre Oslofjord, Midtmeie	Gadus morhua	2015-08-20	Liver	44.8	5.09	0.659							
Indre Oslofjord, Midtmeie	Gadus morhua	2015-08-20	Liver	18.9	2.5	0.293							
Indre Oslofjord, Midtmeie	Gadus morhua	2015-08-20	Liver	40.5	5.81	0.662							

Indre Oslofjord, Midtmeie	Gadus morhua	2015-08-20	Liver	37.3	5.35	0.6
Indre Oslofjord, Midtmeie	Gadus morhua	2015-08-20	Liver	47.2	6.51	0.711
Indre Oslofjord, Midtmeie	Gadus morhua	2015-08-20	Liver	28.6	3.52	0.449
Indre Oslofjord, Midtmeie	Gadus morhua	2015-08-20	Liver	40.4	4.32	0.52
Indre Oslofjord, Midtmeie	Gadus morhua	2015-08-20	Liver	34.2	3.88	0.464
Indre Oslofjord, Midtmeie	Gadus morhua	2015-08-20	Liver	34.6	4.39	0.511
Indre Oslofjord, Midtmeie	Gadus morhua	2015-08-20	Liver	36.1	3.97	0.514
Indre Oslofjord, Midtmeie	Gadus morhua	2015-08-20	Liver	35.7	4.16	0.5
Indre Oslofjord, Midtmeie	Gadus morhua	2015-08-20	Liver	61.8	9.76	1.03
Indre Oslofjord, Midtmeie	Gadus morhua	2016-08-09	Liver		10.2	0.725
Indre Oslofjord, Midtmeie	Gadus morhua	2016-08-09	Liver		4.82	0.445
Indre Oslofjord, Midtmeie	Gadus morhua	2016-08-09	Liver		3.04	0.294
Indre Oslofjord, Midtmeie	Gadus morhua	2016-08-09	Liver		0.967	0.108
Indre Oslofjord, Midtmeie	Gadus morhua	2016-08-09	Liver		14.4	1.3
Indre Oslofjord, Midtmeie	Gadus morhua	2016-08-09	Liver		6.14	0.575
Indre Oslofjord, Midtmeie	Gadus morhua	2016-08-09	Liver		5.85	0.427
Indre Oslofjord, Midtmeie	Gadus morhua	2016-08-09	Liver		3.52	0.38
Indre Oslofjord, Midtmeie	Gadus morhua	2016-08-09	Liver		4.75	0.512
Indre Oslofjord, Midtmeie	Gadus morhua	2016-08-09	Liver		18	1.28
Indre Oslofjord, Midtmeie	Gadus morhua	2016-08-09	Liver		21.5	1.13
Indre Oslofjord, Midtmeie	Gadus morhua	2016-08-09	Liver		18.5	1.77
Indre Oslofjord, Midtmeie	Gadus morhua	2016-08-09	Liver		3.05	0.332
Indre Oslofjord, Midtmeie	Gadus morhua	2016-08-09	Liver		4.77	0.372
Indre Oslofjord, Midtmeie	Gadus morhua	2016-08-09	Liver		9.74	0.844

Table SI-5. Passive sampling-based freely dissolved concentrations of PCBs, HCB and PeCB at three sites on the Norwegian Coast (Oslofjord, Hvaler and Ålesund) monitoring from 2012 to 2016 (ng L^{-1}; grey shaded cells are for data below limits of quantification).

Year	2016	2016	2015	2015	2014	2014	2013	2013
Site	Hvaler	Hvaler	Hvaler	Hvaler	Hvaler	Hvaler	Hvaler	Hvaler
deployment date	25.06.2015	25.06.2015	25.07.2014	25.07.2014	14.10.2013	14.10.2013	15.11.2012	15.11.2012
retrieval date	15.07.2016	15.07.2016	25.06.2015	25.06.2015	25.07.2014	25.07.2014	14.10.2013	14.10.2013
exposure time (d)	386	386	335	335	284	284	333	333
silicone mass (g)	36	32	36	32	32	32	33	31
C_{free} (ng L^{-1})								
CB28	0.0086	0.0053	0.0052	0.0038	0.0133	0.0142	0.0178	0.0141
CB52	0.0073	0.0069	0.0042	0.0031	0.0140	0.0149	0.0057	0.0043
CB101	0.0075	0.0075	0.0058	0.0039	0.0152	0.0163	0.0080	0.0068
CB118	0.0048	0.0051	0.0042	0.0029	0.0156	0.0167	0.0065	0.0068
CB153	0.0120	0.0134	0.0101	0.0080	0.0156	0.0167	0.0138	0.0101
CB138	0.0087	0.0093	0.0045	0.0053	0.0167	0.0178	0.0089	0.0072
CB180	0.0075	0.0057	0.0047	0.0032	0.0173	0.0185	0.0057	0.0014
PeCB	0.0103	0.0107	0.0070	0.0045	0.0239	0.0325	0.0150	0.0145
HCB	0.0339	0.0343	0.0207	0.0152	0.0605	0.0745	0.0568	0.0499
	2016	2016	2015	2015	2014	2014	2013	2013
	Oslofjord	Oslofjord	Oslofjord	Oslofjord	Oslofjord	Oslofjord	Oslofjord	Oslofjord

deployment date	09.06.2015	09.06.2015	22.07.2014	22.07.2014	05.09.2013	05.09.2013	08.10.2012	08.10.2012
retrieval date	13.05.2016	13.05.2016	09.06.2015	09.06.2015	22.07.2014	22.07.2014	05.09.2013	05.09.2013
exposure time (d)	339	339	322	322	320	320	332	332
silicone mass (g)	34	36	34	36	32	32	33	33
C_{free} (ng L^{-1})								
CB28	0.0200	0.0364	0.0214	0.0229	0.0347	0.0337	0.0885	0.0799
CB52	0.0364	0.0643	0.0344	0.0404	0.3147	0.2903	0.1139	0.1042
CB101	0.0163	0.0349	0.0238	0.0324	0.0788	0.0734	0.0768	0.0681
CB118	0.0181	0.0358	0.0204	0.0256	0.0658	0.0554	0.0660	0.0633
CB153	0.0186	0.0358	0.0379	0.0452	0.1043	0.0869	0.0927	0.0842
CB138	0.0155	0.0312	0.0211	0.0291	0.0892	0.0660	0.0627	0.0585
CB180	0.0038	0.0061	0.0058	0.0063	0.0163	0.0113	0.0069	0.0064
PeCB	0.0108	0.0168	0.0078	0.0097	0.0250	0.0283	0.0305	0.0252
HCB	0.0276	0.0478	0.0205	0.0261	0.0787	0.0764	0.0899	0.0800

Year	2016	2016	2015	2015	2014	2014	2013	2013
Site	Ålesund	Ålesund	Ålesund	Ålesund	Ålesund	Ålesund	Ålesund	Ålesund
deployment date	01.07.2015	01.07.2015	05.08.2014	05.08.2014	01.11.2013	01.11.2013	23.11.2012	23.11.2012
retrieval date	11.07.2016	11.07.2016	01.07.2015	01.07.2015	05.08.2014	05.08.2014	01.11.2013	01.11.2013
exposure time (d)	376	376	330	330	277	277	343	343
silicone mass (g)	34	36	34	36	31	32	32	32
C_{free} (ng L^{-1})								
CB28	0.0085	0.0080	0.0050	0.0038	0.0100	0.0069	0.0103	0.0123
CB52	0.0125	0.0105	0.0057	0.0054	0.0070	0.0080	0.0070	0.0077
CB101	0.0065	0.0046	0.0030	0.0024	0.0175	0.0154	0.0075	0.0076
CB118	0.0050	0.0050	0.0030	0.0026	0.0127	0.0089	0.0055	0.0061
CB153	0.0096	0.0086	0.0061	0.0047	0.0252	0.0103	0.0097	0.0097
CB138	0.0066	0.0072	0.0036	0.0032	0.0140	0.0100	0.0065	0.0068
CB180	0.0037	0.0037	0.0020	0.0015	0.0075	0.0076	0.0023	0.0025
PeCB	0.0092	0.0082	0.0058	0.0055	0.0256	0.0176	0.0115	0.0128
HCB	0.0398	0.0355	0.0226	0.0226	0.0810	0.0769	0.0477	0.0529

Table SI-6. Calculated concentrations of PCBs, HCB and PeCB in silicone rubber (SR, AlteSil™) at equilibrium with the water at three sites on the Norwegian Coast (Oslofjord, Hvaler and Ålesund) monitoring from 2012 to 2016 (ng g^{-1}; grey shaded cells are for data below limits of quantification).

Year	2016	2016	2015	2015	2014	2014	2013	2013
C_{sil} (ng g^{-1})	Hvaler	Hvaler	Hvaler	Hvaler	Hvaler	Hvaler	Hvaler	Hvaler
CB28	2.9	1.8	1.8	1.3	4.5	4.8	6.0	4.8
CB52	4.6	4.4	2.7	1.9	8.8	9.4	3.6	2.7
CB101	14.3	14.3	11.0	7.5	29.0	31.0	15.2	12.9
CB118	12.6	13.5	11.1	7.6	41.1	43.9	17.2	18.0
CB153	31.5	35.2	26.6	21.2	41.1	43.9	36.3	26.5
CB138	51.2	54.9	26.5	31.4	98.1	104.7	52.6	42.7
CB180	72.8	55.8	45.7	31.2	169.5	180.9	55.5	13.3
PeCB	0.3	0.3	0.2	0.1	0.8	1.0	0.5	0.5
HCB	3.9	3.9	2.4	1.7	7.0	8.6	6.5	5.7

	2016	*2016*	*2015*	*2015*	*2014*	*2014*	*2013*	*2013*
C_{sil} (ng g^{-1})	Oslofjord	Oslofjord	Oslofjord	Oslofjord	Oslofjord	Oslofjord	Oslofjord	Oslofjord
CB28	6.8	12.3	7.3	7.7	11.8	11.4	30.0	27.1
CB52	22.9	40.6	21.7	25.5	198.6	183.2	71.9	65.8
CB101	31.1	66.6	45.4	61.8	150.1	139.8	146.4	129.8
CB118	47.5	94.2	53.6	67.2	173.0	145.7	173.7	166.6
CB153	49.0	94.2	99.6	118.8	274.2	228.6	243.8	221.5
CB138	91.5	183.9	124.3	171.6	525.1	388.7	369.4	344.7
CB180	37.4	60.0	56.2	61.2	159.4	110.3	67.3	62.4

PeCB	0.3	0.5	0.2	0.3	0.8	0.9	1.0	0.8
HCB	3.2	5.5	2.4	3.0	9.0	8.8	10.3	9.2

	2016	2016	2015	2015	2014	2014	2013	2013
C_{sil} (ng g^{-1})	Ålesund	Ålesund	Ålesund	Ålesund	Ålesund	Ålesund	Ålesund	Ålesund
CB28	2.9	2.7	1.7	1.3	3.4	2.4	3.5	4.2
CB52	7.9	6.6	3.6	3.4	4.4	5.0	4.4	4.8
CB101	12.5	8.8	5.6	4.5	33.4	29.3	14.3	14.4
CB118	13.2	13.0	7.8	6.9	33.4	23.5	14.6	15.9
CB153	25.2	22.5	15.9	12.3	66.4	27.0	25.5	25.4
CB138	39.0	42.3	20.9	19.0	82.5	58.7	38.3	39.8
CB180	36.3	36.6	20.0	14.9	73.8	73.8	22.3	24.3
PeCB	0.3	0.3	0.2	0.2	0.8	0.6	0.4	0.4
HCB	4.6	4.1	2.6	2.6	9.3	8.8	5.5	6.1

Table SI-7. Equivalent concentrations of PCBs, HCB and PeCB in lipids at equilibrium with the water at three sites on the Norwegian Coast (Oslofjord, Hvaler and Ålesund) monitoring from 2012 to 2016 (ng g^{-1} lipid; grey shaded cells are for data below limits of quantification).

	2016	2016	2015	2015	2014	2014	2013	2013
$C_{lip,equiv}$ (ng g^{-1} lip)	Hvaler	Hvaler	Hvaler	Hvaler	Hvaler	Hvaler	Hvaler	Hvaler
CB28	27.7	17.0	16.8	12.2	42.8	45.7	57.0	45.4
CB52	47.8	45.2	27.6	20.2	91.4	97.5	37.2	28.1
CB101	188.8	189.8	145.1	98.9	384.3	410.0	201.7	171.4
CB118	215.8	231.3	189.6	129.2	702.8	749.8	293.8	307.3
CB153	512.3	571.2	432.1	343.6	667.5	712.1	588.9	430.7
CB138	922.4	988.8	476.8	565.4	1767.7	1886.0	947.4	768.9
CB180	1507.8	1154.5	946.5	645.0	3508.8	3743.8	1149.2	275.2

PeCB	2.4	2.5	1.6	1.1	5.6	7.6	3.5	3.4
HCB	36.4	36.9	22.2	16.3	65.0	80.0	61.0	53.6

	2016	2016	2015	2015	2014	2014	2013	2013
$C_{lip,equiv}$ (ng g^{-1} lip)	Oslofjord	Oslofjord	Oslofjord	Oslofjord	Oslofjord	Oslofjord	Oslofjord	Oslofjord
CB28	64.3	116.8	68.7	73.4	111.3	108.3	283.9	256.3
CB52	237.7	420.2	224.9	264.2	2057.1	1897.6	744.6	681.4
CB101	411.2	881.2	601.5	818.5	1987.8	1851.4	1938.3	1718.4
CB118	811.8	1611.0	916.7	1149.3	2959.0	2490.9	2970.2	2848.5
CB153	795.6	1530.0	1616.8	1929.1	4453.4	3712.0	3959.1	3597.0
CB138	1648.8	3313.6	2239.5	3092.1	9461.8	7004.8	6657.4	6212.1
CB180	774.9	1242.2	1163.6	1267.5	3299.5	2283.4	1392.6	1292.3
PeCB	2.5	3.9	1.8	2.3	5.8	6.6	7.1	5.9
HCB	29.6	51.3	22.0	28.0	84.4	82.0	96.5	85.9

	2016	2016	2015	2015	2014	2014	2013	2013
$C_{lip,equiv}$ (ng g^{-1} lip)	Ålesund	Ålesund	Ålesund	Ålesund	Ålesund	Ålesund	Ålesund	Ålesund
CB28	27.2	25.6	15.9	12.2	32.0	22.3	32.9	39.3
CB52	81.6	68.8	37.3	35.6	45.8	52.3	45.6	50.1
CB101	164.9	116.3	74.5	59.6	441.7	387.9	188.8	191.2
CB118	226.0	222.7	133.5	117.7	571.0	402.3	248.9	272.4
CB153	408.8	365.3	258.8	200.0	1077.9	439.1	413.6	413.0
CB138	703.1	763.0	376.7	342.5	1487.4	1056.9	690.8	716.8
CB180	751.3	757.1	414.6	309.0	1527.1	1528.4	461.9	502.8
PeCB	2.1	1.9	1.3	1.3	6.0	4.1	2.7	3.0
HCB	42.7	38.2	24.3	24.3	87.0	82.5	51.2	56.8

Table SI-8. Lipid-water partition coefficients for pentachlorobenzene (PeCB) and hexachlorobenzene (HCB).

Compound	$\log K_{sr\text{-}w}$ (L kg^{-1})[a]	$K_{lip\text{-}sr}$ (kg kg^{-1})[a]	$\log K_{lip\text{-}w}$ (L kg^{-1})	$K_{lip\text{-}w}$ (L kg^{-1})	$K_{lip\text{-}w,HCB}/K_{lip\text{-}w,PeCB}$
PeCB	4.62	7.38	5.49	307650	
HCB	5.05	9.35	6.02	1049087	3.4
CB28	5.53	9.47	6.55	3568029	3.4[b]
CB52	5.81	10.36	6.91	8167526	7.8[b]
CB101	6.28	13.24	7.31	20483703	20[b]
CB118	6.42	17.10	7.68	47423732	45[b]
CB138	6.77	16.20	7.97	94273869	90[b]
CB153	6.72	18.02	8.07	118291602	113[b]
CB180	6.99	20.70	8.38	239227672	228[b]

[a]For AlteSil silicone rubber[1, 7]

[b]Benchmarking for PCB is based on HCB ($K_{lip\text{-}w,\,PCB}/K_{lip\text{-}w,\,HCB}$)

Table SI-9. Median cod-water activity ratios for sampling locations (Andøya, Bear Island and Jan Mayen) where data from the silicone rubber passive samplers (denominator in the activity ratio) were below limits of quantification.

	Andøya	Bear Island	Jan Mayen
CB28		0.32 (0.16-0.95)	
CB52	0.42 (0.30-2.85)	0.20 (0.09-1.07)	1.47 (0.76-2.39)
CB101	0.38 (0.19-2.68)	0.09 (0.04-0.76)	0.79 (0.44-1.3)
CB118	0.16 (0.08-1.49)	0.05 (0.01-0.33)	0.42 (0.25-0.81)
CB138	0.11 (0.06-0.93)	0.03 (0.01-0.24)	0.30 (0.17-0.56)
CB153	0.11 (0.06-1.02)	0.02 (0.01-0.23)	0.27 (0.15-0.47)
CB180	0.01 (0.01-0.09)	0.002 (0.001-0.02)	0.03 (0.02-0.06)

Table SI-10. Median cod-water activity ratios for sampling locations (Hvaler and Ålesund) where data from the silicone rubber passive samplers (denominator in the activity ratio) were below limits of quantification.

	Hvaler			Ålesund
Year	2014	2015	2016	2014
CB28	0.25 (0.13-0.38)			
CB52	0.17 (0.16-0.18)			3.0 (0.74-7.4)
CB101	0.11 (0.04-0.13)	0.33 (0.13-0.87)		
CB118	0.20 (0.16-0.21)	1.04 (0.31-1.9)	0.55 (0.27-0.98)	
CB138	0.17 (0.12-0.19)	1.02 (0.32-1.5)		
CB153	0.69 (0.62-0.88)			
CB180	0.03 (0.026-0.042)	0.24 (0.06-0.29)	0.08 (0.03-0.11)	0.16 (0.02-0.55)

References

1. Smedes, F.; Geertsma, R. W.; Zande, T. v. d.; Booij, K., Polymer− water partition coefficients of hydrophobic compounds for passive sampling: Application of cosolvent models for validation. *Environmental Science & Technology* **2009,** *43*, (18), 7047-7054.
2. Booij, K.; Smedes, F., An improved method for estimating in situ sampling rates of nonpolar passive samplers. *Environmental science & technology* **2010,** *44*, (17), 6789-6794.
3. Rusina, T. P.; Smedes, F.; Koblizkova, M.; Klanova, J., Calibration of silicone rubber passive samplers: experimental and modeled relations between sampling rate and compound properties. *Environmental science & technology* **2009,** *44*, (1), 362-367.
4. Allan, I. J.; Harman, C.; Ranneklev, S. B.; Thomas, K. V.; Grung, M., Passive sampling for target and nontarget analyses of moderately polar and nonpolar substances in water. *Environmental toxicology and chemistry* **2013,** *32*, (8), 1718-1726.
5. Allan, I. J.; Vrana, B.; de Weert, J.; Kringstad, A.; Ruus, A.; Christensen, G.; Terentjev, P.; Green, N. W., Passive sampling and benchmarking to rank HOC levels in the aquatic environment. *Scientific reports* **2021,** *11*, (1), 1-12.
6. Lyche, J. L.; Nøstbakken, O. J.; Berg, V., EU Water Framework-directive Priority Contaminants in Norwegian Freshwater Fish.
7. Smedes, F.; Rusina, T. P.; Beeltje, H.; Mayer, P., Partitioning of hydrophobic organic contaminants between polymer and lipids for two silicones and low density polyethylene. *Chemosphere* **2017,** *186*, 948-957.
